# Association of apolipoprotein E variants on Alzheimer's disease in Latin America: A systematic review and meta‐analysis

**DOI:** 10.1002/alz.71224

**Published:** 2026-02-22

**Authors:** Paulina Orellana, Ariel Caviedes, Liset Gonzalez, Carolina Ochoa‐Rosales, Danilo Carmona, Carolina González‐Silva, Hernán Hernández, Gabrielle B. Britton, Alcibiades E. Villarreal, Victoria Campos, Hugh Hendrie, Juliana Acosta‐Uribe, Stefanie D. Pina‐Escudero, Jennifer S. Yokoyama, Natalia Vilor‐Tejedor, Mario Parra, Hernando Santamaria‐García, Agustín Ibañez, Rolando de la Cruz, Claudia Duran‐Aniotz

**Affiliations:** ^1^ Latin American Brain Health Institute (BrainLat) Universidad Adolfo Ibanez Santiago Chile; ^2^ Department of Human Genetics Radboud University Medical Center Nijmegen the Netherlands; ^3^ Instituto de Investigaciones Científicas y Servicios de Alta Tecnología (INDICASAT AIP) Panamá Panamá; ^4^ Laboratorio Experimental de Enfermedades Neurodegenerativas Instituto Nacional de Neurología y Neurocirugía Manuel Velasco Suárez Ciudad de México Mexico; ^5^ School of Medicine Indiana University Indianapolis Indiana USA; ^6^ Grupo de Neurociencias de Antioquia (GNA) Facultad de Medicina Universidad de Antioquia Medellín Colombia; ^7^ Neuroscience Research Institute and Department of Molecular, Cellular and Developmental Biology University of California, Santa Barbara Santa Barbara California USA; ^8^ Weill Institute for Neurosciences University of California, San Francisco San Francisco California USA; ^9^ Global Brain Health Institute (GBHI) University of California, San Francisco (UCSF) San Francisco California USA; ^10^ Global Brain Health Institute (GBHI) Trinity College Dublin (TCD) Dublin Ireland; ^11^ Memory and Aging Center University of California, San Francisco San Francisco California USA; ^12^ Institute for Risk Assessment Sciences (IRAS) Department of Veterinary Medicine Utrecht University Utrecht the Netherlands; ^13^ Barcelonaβeta Brain Research Center (BBRC) Pasqual Maragall Foundation Barcelona Spain; ^14^ Department of Human Genetics Radboud UMC Nijmegen the Netherlands; ^15^ School of Psychological Sciences and Health University of Strathclyde Glasgow UK; ^16^ Pontificia Universidad Javeriana (PhD Program in Neuroscience) Bogotá San Ignacio Colombia; ^17^ Center of Memory and Cognition Intellectus Hospital Universitario San Ignacio Bogotá San Ignacio Colombia; ^18^ Cognitive Neuroscience Center (CNC) Universidad de San Andrés, & CONICET Buenos Aires Argentina; ^19^ Department of Biophysics, School of Medicine Istanbul Medipol University Istanbul Turkey; ^20^ Faculty of Engineering and Sciences Universidad Adolfo Ibáñez Santiago Chile

**Keywords:** Alzheimer's disease, Caribbean, genetic risk factors, Latin America, meta‐analysis, systematic review

## Abstract

The apolipoprotein E (*APOE*) ε4 allele represents the strongest genetic risk factor for Alzheimer's disease (AD), but its role in genetically diverse Latin American and Caribbean (LAC) populations is underexplored. We conducted a meta‐analysis of 35 studies from 11 LAC countries, encompassing 3206 patients with AD and 5515 controls. The ε4 allele demonstrated significant association with increased AD risk (odds ratio [OR] = 3.25, 95% confidence interval [2.82–3.76]), while ε3 showed lower odds (0.42, [0.37–0.48]). Homozygous ε4/ε4 carriers had elevated risk (6.84, [5.09–9.19]), and heterozygous ε3/ε4 carriers showed moderate risk (2.59, [2.31–2.91]). Country‐level analyses revealed variability, with Ecuador showing the highest OR for ε4/ε4 (13.29, [1.56–113.4]). These results confirm *APOE* ε4 as a major AD risk factor in LAC populations and highlight regional differences relevant to precision medicine.

## Highlights

- This study is the largest regional apolipoprotein E meta‐analysis on Alzheimer's disease (AD) risk across Latin America.
- The ε4 allele is associated with AD risk, but with regional variability across Latin American and Caribbean (LAC) countries.
- The ε3 allele showed a protective effect in LAC populations (pooled odds ratio 0.42).
- The ε2 allele us not associated with protection in LAC, diverging from findings in other regions.
- Findings underscore the need for region‐specific dementia risk estimates.

## 1 INTRODUCTION

### 1.1 Alzheimer's disease in Latin American and Caribbean countries

Alzheimer's disease (AD) represents one of the most pressing public health challenges in the landscape of public health in Latin American and Caribbean (LAC) countries.^1^, ^2^ It is a leading cause of disability among older adults, and profoundly impacts the quality of life of patients, families, and caregivers, imposing substantial socioeconomic burdens on health‐care systems and society.^1^, ^2^ The prevalence of AD in LAC countries reaches 8.5% in people > 65 years old with projections suggesting this figure will triple by the year 2050.^3^, ^4^ Notably, this prevalence surpasses rates documented in Europe and the United States.^4^, ^5^ These regional disparities in prevalence may be partially attributed to distinct genetic and environmental factors, underscoring the critical need for region‐specific research initiatives.^6^ Despite this alarming epidemiological trend, the region remains critically underrepresented in AD research,^7^, ^8^ thereby impeding the development of culturally appropriate interventions and prevention strategies that consider the region's unique genetic landscape. Because genetic association estimates depend on genotypes distribution and the population structure, apparent differences across regions can be driven by genotype frequencies, ancestry composition, sampling frames, and diagnostic/study‐design heterogeneity, rather than population‐specific differences in the biology of the encoded proteins.^9^, ^10^, ^11^ This consideration is particularly relevant in LAC, where recruitment contexts and admixture proportions vary substantially across countries and studies.^3^, ^12^

### 1.2 Apolipoprotein E gene and AD

Apolipoprotein E (*APOE*) stands as one of the most well‐studied genetic risk factors for AD.^13^, ^14^ This gene maps to chromosome 19q13.32, and encodes a major apolipoprotein, key to lipids transport, metabolism, and homeostasis.^14^ There are three common *APOE* alleles (ε2, ε3, and ε4) that originate three distinct isoforms: apoE2 (Cys112, Cys158), apoE3 (Cys112, Arg158), and apoE4 (Arg112, Arg158). They differ by amino acid substitutions at positions 112 and 158,^13^ resulting in structural and functional differences across *APOE* isoforms. These variations confer different lipid and receptor binding capacities, playing a pivotal role in brain lipid metabolism and cholesterol transport.^13^, ^14^ In the context of AD pathogenesis, *APOE* modulates critical disease mechanisms including amyloid beta (Aβ) metabolism, neuroinflammation, neuronal repair processes, and blood–brain barrier integrity.^15^, ^16^, ^17^

In epidemiologic studies, ε3 is typically treated as the most common reference/baseline allele or genotype for comparisons of AD odds. Relative to ε3/ε3, ε4 carriage is consistently associated with higher odds of late‐onset AD across many populations, whereas ε2 is often associated with lower odds, although effect sizes vary by study and population.^11^, ^14^, ^15^, ^18^, ^19^

### 1.3 *APOE* variability across populations

Globally, *APOE* allele frequencies are estimated at ≈ 8% for ε2, 78% for ε3, and 14% for ε4.^15^ The ε2 allele has been associated with lower odds of AD relative to ε3 in multiple studies, whereas the ε4 allele significantly elevates the odds of developing late‐onset AD (LOAD).^13^, ^14^, ^15^ Notably, the ε4 allele frequency increases to ≈ 40% among AD patients,^15^ with heterozygous carriers (ε3/ε4) facing a 3‐ to 4‐fold higher risk and homozygous carriers (ε4/ε4) experiencing a 9‐ 15‐fold increased risk.^13^, ^14^ *APOE* allele distribution demonstrates considerable variation across global populations,^16^, ^17^ with African and Oceanian populations exhibiting higher frequencies of both ε2 and ε4 alleles, while Indian and Asian populations show higher frequencies of the ε3 allele.^20^ A comprehensive systematic review covering the period from 1985 to 2010 revealed that Northern Europeans demonstrate the highest ε4 prevalence among AD patients, whereas Asian and Southern European populations exhibit the lowest frequencies.^21^ Similarly, in China, the ε4 frequency reaches 13.1%, with a disproportionately higher representation of ε4 carriers among patients diagnosed with mild cognitive impairment (MCI) and AD.^22^ Importantly, the association between AD and the ε4 allele exhibits population‐specific variations. The relationship appears weaker or even paradoxical in African Americans and Hispanics, while Japanese populations demonstrate a stronger association than Caucasians.^11^, ^23^ Within Latino populations, *APOE* allele frequencies and their association with AD demonstrate notable variability.^24^, ^25^ A recent meta‐analysis revealed that while the ε4 allele significantly increases AD risk in South American subgroups, this association is not consistently observed in other Hispanic populations.^26^

The LAC population is characterized by extensive genetic admixture, resulting from centuries of intermingling among European, Indigenous, and African ancestral groups.^27^, ^28^ This unique genetic heritage, coupled with marked social and environmental inequalities, likely influences the effect of the *APOE* ε4 allele on AD risk in ways that differ from patterns observed in other global populations. Despite extensive research on *APOE* and AD associations, most studies have focused predominantly on US and European cohorts, leaving LAC populations critically underrepresented in the literature. This research gap poses significant risks, as extrapolating findings from these predominantly studied groups may overlook crucial regional differences in disease etiology, progression, and therapeutic responses. Moreover, studies conducted in LAC remain scarce and fragmented, limiting the ability to generate contextually relevant evidence to inform prevention, diagnosis, and care strategies. To address these gaps, our study systematically reviews and meta‐analyzes 35 studies from 11 LAC countries, comprising 3206 individuals with AD and 5515 controls (total *n* = 8721 participants). We evaluated the association of *APOE* alleles and genotypes with AD prevalence across diverse regional contexts. Our central hypothesis was that, given the heterogeneity across LAC populations, observed *APOE–*AD association estimates in LAC may differ from other regions primarily due to differences in genotype frequencies, ancestry composition, sampling frames, and diagnostic/study‐design heterogeneity rather than population‐specific differences in *APOE* isoform biology. Therefore, we systematically reviewed and meta‐analyzed studies from LAC to quantify the association of *APOE* alleles and genotypes with AD and to describe allele and genotype frequency patterns across included studies.

## 2 METHODOLOGY

### 2.1 Literature search strategy

To identify potentially eligible studies, the Preferred Reporting Items for Systematic Reviews and Meta‐Analyses (PRISMA) checklist and PRISMA statement were followed.^29^ An exhaustive search was performed through PubMed, Scielo, and Scopus databases for relevant studies using the following terms: ((Alzheimer's disease) OR (AD)) AND (*APOE*)) OR (Apolipoprotein E)) AND ((Argentina) OR (Bolivia) OR (Brazil) OR (Chile) OR (Colombia) OR (Costa Rica) OR (Cuba) OR (Ecuador) OR (El Salvador) OR (French Guayana) OR (Granada) OR (Guatemala) OR (Guyana) OR (Honduras) OR (Haiti) OR (Jamaica) OR (Mexico) OR (Nicaragua) OR (Paraguay) OR (Panama) OR (Peru) OR (Caribbean) OR (Puerto Rico) OR (Dominican Republic) OR (Surinam) OR (Uruguay) OR (Venezuela) OR (Latin America)). No restrictions on language or publication year were applied. Cross‐references retrieved other relevant references of identified studies.

### 2.2 Inclusion/exclusion criteria

Two independent authors (P.O. and A.C.) conducted a parallel selection of relevant articles based on the following criteria. Studies were included if they: were conducted in LAC populations, verified by confirming that the cohorts described in the methodology were recruited from institutions or research centers located within LAC countries, or included participants of LAC origin; were articles that investigated the association between *APOE* alleles and AD risk; used case–control or cohort study designs; included alleles and genotype data for both case and control groups; provided sufficient data for calculating odds ratios (ORs) and 95% confidence intervals (CIs); and demonstrated Hardy–Weinberg equilibrium (HWE) in genotype distribution among control groups. Studies were excluded if they: contained redundant data already reported in other publications (i.e., studies with overlapping populations); relied on case reports, reviews, or abstracts; presented populations outside of LAC; were based on animal models; lacked genotype frequency information; or failed to confirm to HWE in the control group.

### 2.3 Data extraction

**FIGURE 1:**
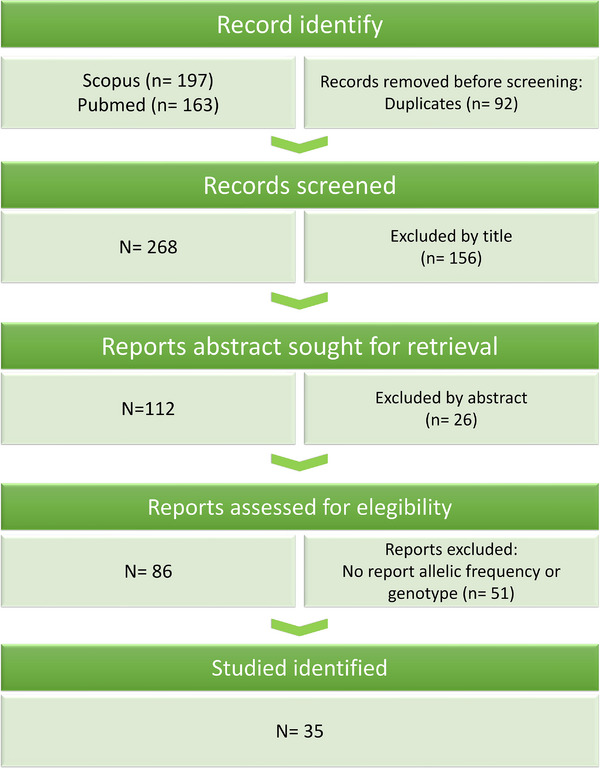
Preferred Reporting Items for Systematic Reviews and Meta‐Analyses systematic review structure. The search of PubMed, Scopus, and SciElo databases yielded 360 citations. After the title review, 156 studies were excluded, and 26 abstracts did not meet the criteria. A further examination of 86 full texts led to the exclusion of 51 studies, resulting in 35 articles selected for analysis.

The search in the PubMed, Scopus, and SciElo databases yielded 268 citations. After reviewing the titles, 156 studies were discarded, and 26 abstracts did not meet the inclusion criteria. A detailed review of the full texts of the remaining 86 citations was performed. To supplement allele frequency and genotype data when unavailable, we contacted study authors; however, most requests were unsuccessful, with data obtained via e‐mail in only three cases. Ultimately, 51 studies were excluded for not meeting the inclusion criteria. No unpublished relevant studies were identified, resulting in a final selection of 35 articles for analysis. Data extraction included the author's name, publication year, participant characteristics (ethnicity, age, and gender/sex), genotyping methods, AD diagnostic criteria, sample size for both cases and controls, allelic frequencies, and genotype counts. In cases in which authors did not report the absolute or relative *APOE* allele frequency, we estimated it based on the reported *APOE* genotypes. Allelic and genotype frequencies for *APOE* were calculated by first tabulating the observed *APOE* genotypes (ε2/ε2, ε2/ε3, ε3/ε3, ε3/ε4, and ε4/ε4). The allele frequencies for ε2, ε3, and ε4 were determined by adding twice the number of individuals with the homozygous genotype for a given allele (e.g., ε2/ε2) to the number of individuals with heterozygous genotypes containing the allele (e.g., ε2/ε3 and ε2/ε4 for ε2) and dividing this sum by twice the total number of individuals. This method was applied consistently to calculate the frequencies of ε3 and ε4 alleles. Genotype frequencies were calculated by dividing the number of individuals with each genotype by the total sample size. These frequencies were then used to compute individual and pooled standardized effect size estimates, assess potential sources of bias, and generate all figures. Any discrepancies were resolved through consensus with a third author (C.G.S.). The article selection procedure is illustrated in the flowchart shown in Figure 1. We additionally extracted (or derived, when necessary) country‐level allele and genotype distributions in cases and controls to explicitly describe frequency patterns across LAC studies and contextualize between‐study heterogeneity.

### 2.4 Statistical analyses

The strength of relationship between *APOE* ε2, ε3, ε4 alleles; ε2/ε2, ε2/ε3, ε2/ε4, ε3/ε3, ε3/ε4, and ε4/ε4 genotypes; and AD susceptibility was evaluated using crude ORs with 95% CIs. For allele‐based analyses, ORs were computed from allele counts in cases versus controls (i.e., allele enrichment/depletion), which represents a case–control comparison rather than an intrinsic “effect” of an allele in isolation. Accordingly, OR < 1 for ε3 is interpreted as relative depletion of ε3 among cases (given enrichment of non‐ε3 alleles in cases).

**FIGURE 2:**
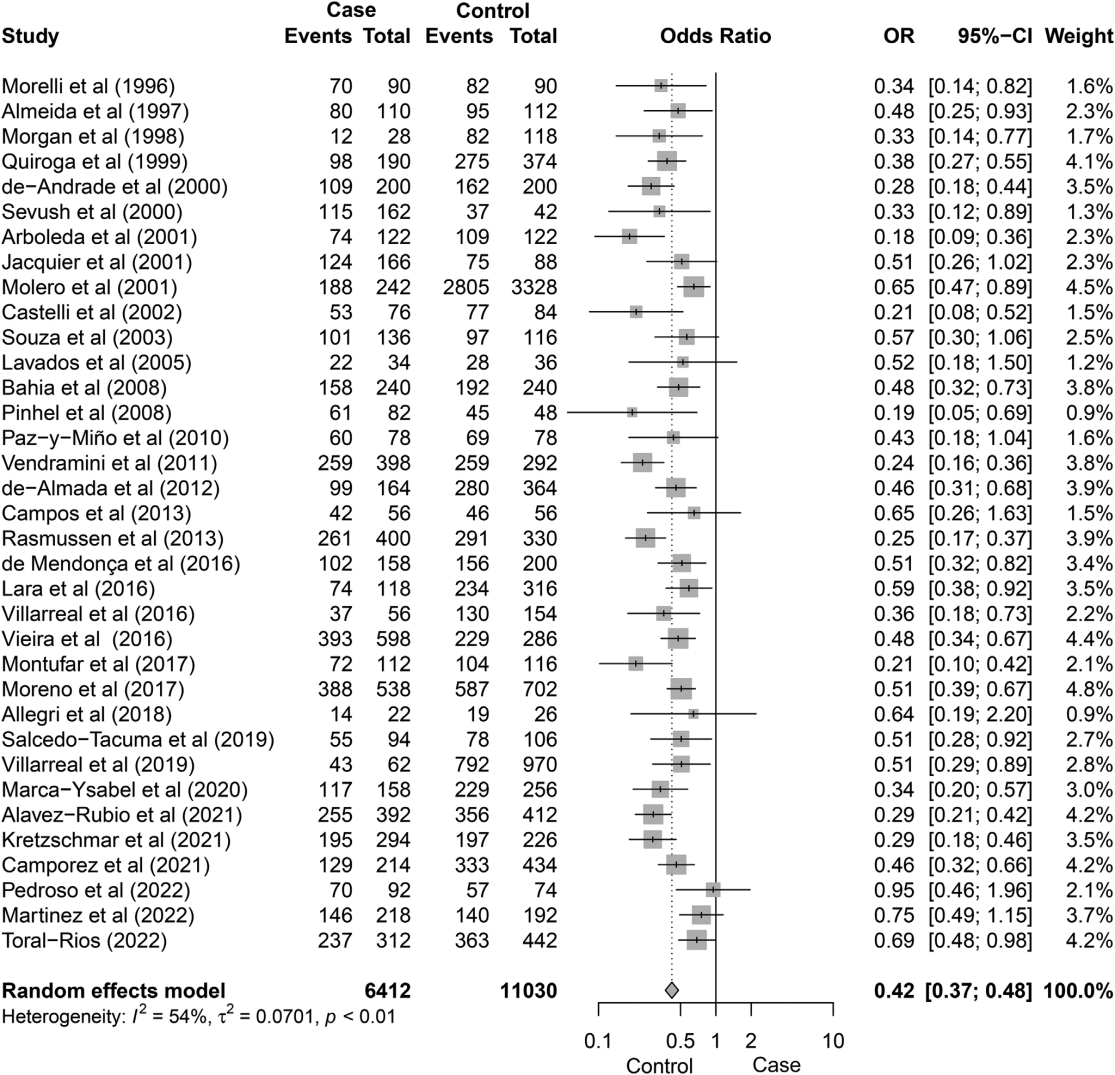
Forest plot displaying the results of a meta‐analysis evaluating the association between the *APOE* ε3 allele and AD across 35 studies. Each study's OR and 95% CI are shown, with square markers representing individual study estimates, sized according to their relative weight in the meta‐analysis. The pooled OR under the random‐effect model is 0.42 (95% CI: 0.37–0.48), indicating an inverse association (OR < 1) in this allele‐count case–control meta‐analysis, consistent with relative depletion of ε3 among AD cases given enrichment of ε4 in cases. Moderate heterogeneity (*I* ^2^ = 54%) was observed, suggesting some variability across studies. The overall results are visually summarized with the diamond at the bottom of the plot, reflecting the combined effect estimate and CI. τ^2^ = between‐study variance; *P* = *P* value. AD, Alzheimer's disease; *APOE*, apolipoprotein E; CI, confidence interval; OR, odds ratio.

**FIGURE 3:**
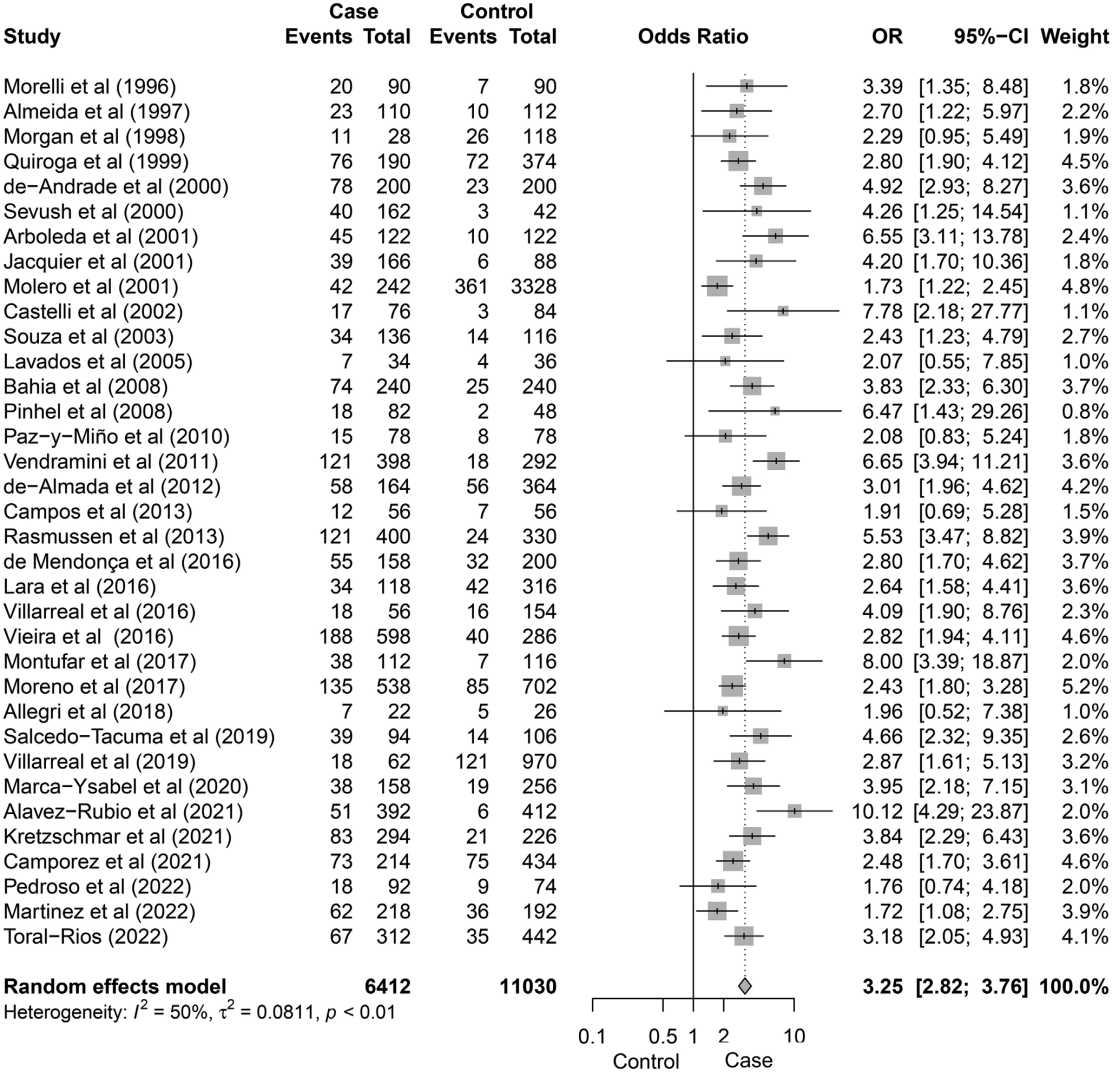
Forest plot displaying the results of a meta‐analysis evaluating the association between the *APOE* ε4 allele and AD across 35 studies. Each study's OR and 95% CI are shown, with square markers representing individual study estimates, sized according to their relative weight in the meta‐analysis. The pooled OR, calculated using a random‐effect model, is 3.25 (95% CI [2.82–3.76]), indicating a significant association between the *APOE* ε4 allele and higher odds of AD. Moderate heterogeneity was observed (*I* ^2^ = 50%), suggesting some variability across studies. The overall results are visually summarized with the diamond at the bottom of the plot, reflecting the combined effect estimate and CI. τ^2^ = between‐study variance; *P* = *P* value. AD, Alzheimer's disease; *APOE*, apolipoprotein E; CI, confidence interval; OR, odds ratio.

Subsequently, stratified analyses by country were performed. Because not all studies analyzed the three alleles (ε2, ε3, ε4), a total of 31 studies were included for the ε2 allele and 35 for the ε3 and ε4 allele, as detailed in the Results section (Figures S1 in supporting information and Figures 2 and 3). In addition, we evaluated the association of the ε2 and ε4 alleles using the ε3 allele as reference/baseline allele, consistent with common practice in *APOE* epidemiologic studies (Figures S2 and S3 in supporting information). All statistical analyses were performed using R software (version 4.3.1). For the meta‐analysis, the R‐packages meta,^30^ metafor,^31^ and metaviz^32^ were used to calculate ORs with 95% CIs. For clarity, we referred to “allele‐count case–control ORs” when estimating enrichment/depletion of each allele in cases versus controls.

### 2.5 Additional analysis

Country‐specific analyses were performed for *APOE* alleles (ε2, ε3, and ε4) and genotypes (ε2/ε2, ε2/ε3, ε2/ε4, ε3/ε3, ε3/ε4, and ε4/ε4). Fixed‐ or random‐effects models were applied based on heterogeneity levels assessed by the *I* ^2^ statistic. We reported both pooled and country‐stratified estimates to emphasize that heterogeneity across LAC is expected to reflect differences in genotype frequencies, ancestry composition, sampling frames, and diagnostic/study‐design variability rather than population‐specific differences in *APOE* isoform biology. Studies with insufficient genotype frequency were excluded from the analysis. This approach enabled the evaluation of country‐specific AD risk across Argentina, Brazil, Chile, Colombia, Ecuador, Mexico, Panama, and Venezuela.

### 2.6 Assessment of heterogeneity

An *X* ^2^‐based Cochran Q test and Higgins *I* ^2^ statistic were used to evaluate the between‐study heterogeneity of the studies. *I* ^2^ values of > 50% indicate heterogeneity among studies, and a *P* value of < 0.05 was considered significant.^33^, ^34^ To carry out the meta‐analysis, a fixed‐effect model (the Mantel–Haenszel method) was adopted if there was no significant heterogeneity (*I* ^2^ < 50%). Otherwise, the random effect model (the DerSimonian and Laird method) was used. The stability of the results was assessed using a sensitivity “leave‐one‐out” meta‐analysis, which omitted a single study each time to evaluate the influence of a single study on the pooled OR. When heterogeneity was substantial, we prioritized random‐effects estimates and interpreted pooled results as average associations across diverse study contexts rather than as a single universal effect size.

### 2.7 Risk of bias assessment/quality assessment

Funnel plots were used to assess publication bias, plot the effect size, and measure the precision of the effect size. The Begg and Egger tests^35^ were performed to determine the funnel asymmetry. An asymmetric plot (*P* < 0.05) was suggestive of possible publication bias.

## 3 RESULTS

### 3.1 Study characteristics

A total of 35 studies were included following PRISMA guidelines, comprising 8376 participants (3206 with AD and 5515 controls) from 11 LAC countries: 2 studies from Argentina,^36^, ^37^ 13 from Brazil,^38^, ^39^, ^40^, ^41^, ^42^, ^43^, ^44^, ^45^, ^46^, ^47^, ^48^, ^49^, ^50^ 2 from Chile,^51^, ^52^ 4 from Colombia,^53^, ^54^, ^55^, ^56^ 1 from Cuba,^57^ 2 from Ecuador,^58^, ^59^ 1 from Jamaica,^60^ 4 from Mexico,^61^, ^62^, ^63^, ^64^ 2 from Panama,^65^, ^66^ 1 from Peru,^67^ and 3 from Venezuela.^68^, ^69^, ^70^ Only three studies reported sex‐specific analyses.^42^, ^69^, ^70^ The full list of studies and details on diagnostic criteria and genotyping approaches are provided in Table 1.

**TABLE 1 alz71224-tbl-0001:** Characteristics of included studies.

First author	Year	Country	Age (case/control)	Sex, *n* (F%) (case/control)	Genotyping methods	Diagnostic criteria	Case/control
Allegri^32^	2018	Argentina	72.3 ± 14.4/69.2 ± 5.8	8(44)/21(49)	NA	MMSE‐RAVLT‐BNT‐TMT‐CDR‐NPI‐GDS	43/18
Morelli^33^	1996	Argentina	74.7 ± 5.5/71.9 ± 7.2	NA	PCR‐RFLP	NINCDS‐ADRDA	45/45
Almeida^34^	1997	Brazil	68.3 ± 2.4/75 ± 1.3	35(62.8)/39(69.6)	NA	NINCDS‐ADRDA	55/56
Bahia^35^	2008	Brazil	75.2 ± 9.2/72.5 ± 8.6	82(68.3)/76(63.3)	PCR‐RFLP	NINCDS‐ADRDA, MMSE	120/120
Camporez^36^	2021	Brazil	82.3 ± 7.6/81.2 ± 9.9	75(68.8)/164(73.5)	Taqman	NINCDS/ADRDA, DSM‐IV, MMSE	109/223
de‐Almada^37^	2012	Brazil	82.2 ± 7.5/78.3 ± 8.3	56(68.3)/131(72)	PCR‐RFLP	MMSE, CDR	82/182
de‐Andrade^38^	2000	Brazil	72.3 ± 3.3/57 ± 13.1	NA	PCR‐RFLP	NINCDS‐ADRDA	100/100
Kretzschmar^39^	2021	Brazil	75.6(60–90)/70.8(60–99)	95(64.6)/85(75.2)	Taqman	MMSE	147/113
Lara^40^	2016	Brazil	NA	36(61)/94(59.5)	PCR‐RFLP	DSM‐IV	59/158
Pedroso^41^	2022	Brazil	79.7 ± 6.9/71.8 ± 7.3	27(56)/30(81)	Taqman	DSM‐V, CDR	46/37
Pinhel^42^	2008	Brazil	81.1 ± 7.3/70.6 ± 5.1	32(77.5)/18(75)	PCR‐RFLP	NINCDS‐ADRDA	41/24
Rasmussen^43^	2013	Brazil	75.3 ± 7.9/71.7 ± 8.1	130(65)/110(66.7)	PCR‐RFLP	NINCDS‐ADRDA, MMSE, DSM‐IV	200/165
Souza^44^	2003	Brazil	71.5/70.0	29(42.6)/30(51.7)	PCR‐RFLP	NINCDS‐ADRDA	68/58
Vendramini^45^	2011	Brazil	73.8 ± 8/71.3 ± 9	131(65.8)/93(63.7)	PCR‐RFLP	NINCDS‐ADRDA	199/146
Vieira^46^	2016	Brazil	79(74–83)/76(71–82)	200(67)/96(67)	Taqman	NINCDS‐ADRDA	299/143
Lavados^47^	2005	Chile	71.4 ± 6.6/67.5 ± 5.9	9(52.9)/11(61.1)	PCR‐RFLP	NINCDS‐ADRDA	17/18
Quiroga^48^	1999	Chile	80.7 ± 1.5/78.2 ± 1	NA	Isoelectric and immunoblotting	NINCDS‐ADRDA, DSM‐III	95/187
Arboleda^49^	2001	Colombia	72.4 ± 9.0/72.4 ± 8.7	45(73.8)/45(73.8)	PCR‐RFLP	MMSE	61/61
Jacquier^50^	2001	Colombia	73.3 ± 9.5/65.8 ± 7.2	47(56.6)/29(65.9)	PCR‐RFLP	NINCDS‐ADRDA, MMSE	83/44
Moreno^51^	2017	Colombia	75.5 ± 7.2/71 ± 7.1	213(76.1)/264(73.9)	PCR‐RFLP	DSM‐IV, MMSE	280/357
Salcedo‐Tacuma^52^	2019	Colombia	72.2 ± 7/72.1 ± 7	25(50)/24(48)	Taqman	DSM‐IV	47/53
Sevush^53^	2000	Cuba	NA	NA	PCR‐RFLP	NINCDS‐ADRDA	80/21
Montufar^54^	2017	Ecuador	78 ± 6.3/76.6 ± 7.1	30(53.6)/31(53.4)	qPCR FRET	NIA‐AA criteria, MMSE	56/58
Paz‐y‐Miño^55^	2010	Ecuador	78.4 ± 6.8/77.9 ± 6.5	27(69.2)/27(69.2)	PCR‐RFLP	NA	39/39
Morgan^56^	1998	Jamaica	82.2 ± 5.6/79.5 ± 6.5	NA	PCR‐RFLP	NINCDS‐ADRDA, DSM‐III	14/59
Alavez‐Rubio^57^	2021	Mexico	77 ± 11/70 ± 11	128(65.3)/130(63.7)	Taqman	NINCDS‐ADRDA, NINDS‐AIREN, MMSE, DMS‐IV	196/206
Campos^58^	2013	Mexico	NA	NA	NA	NA	28/28
Castelli^59^	2002	Mexico	82 ± 8/83 ± 7	24(63.2)/30(71.4)	PCR‐RFLP	DSM‐IV, MMSE	38/42
Toral‐Rios^60^	2022	Mexico	76.1 ± 8.8/73.6 ± 8.5	106(67.9)/167(75.6)	Taqman	NINCDS‐ADRDA	156/221
Villarreal^61^	2016	Panamá	82.1 ± 8.8/76.7 ± 6.9	24(77.4)/120(64.9)	NA	NINCDS‐ADRDA, MMSE	31/185
Villarreal^62^	2019	Panamá	81.9 ± 7.7/70.8 ± 8.3	23(74.2)/156(32.2)	NA	NA	31/485
Marca‐Ysabel^63^	2021	Peru	72.3 ± 8.4/75.0 ± 6.6	53(67)/76 (59.1)	PCR‐RFLP	NINCDS‐ADRDA	79/128
de Mendonça^64^	2016	Venezuela	70 ± 10/71 ± 10	NA	PCR‐RFLP	NINCDS‐ADRDA	79/100
Martinez^65^	2022	Venezuela	83.6 ± 28/68.7 ± 8	79(73)/59(61)	Seeplex ApoE	MMSE	108/96
Molero^66^	2001	Venezuela	78.1 ± 9.0/66.5 ± 8.4	101(83.5)/1109(66.6)	PCR‐RFLP	NINCDS‐ADRDA	121/1664

*Note*: Summary of the selected studies for the meta‐analysis, including the first author, publication year, and country of each study. It also presents demographic details, such as the age and sex distribution (*n* and percentage of females) in both case and control groups. The genotyping methods, diagnostic criteria, and the number of cases and controls are outlined.

Abbreviations: BNT, Boston Naming Test; CDR, Clinical Dementia Rating; DSM, Diagnostic and Statistical Manual of Mental Disorders; GDS, Global Deterioration Scale; MMSE, Mini‐Mental State Examination; NA, not available; NIA‐AA, National Institute on Aging and the Alzheimer's Association; NINCDS‐ADRDA, National Institute of Neurological and Communicative Disorders and Stroke and the Alzheimer's Disease and Related Disorders Association; NINDS‐AIREN, National Institute of Neurological Disorders and Stroke and the Association Internationale pour la Recherche et l'Enseignement en Neurosciences; NPI, neuropsychiatric inventory; PCR‐RFLP, polymerase chain reaction restriction fragment length polymorphism; qPCR FRET, quantitative polymerase chain reaction fluorescence resonance energy transfer; RALVT, Rey‐ Auditory Verbal Learning Test; TMT, Trail Making Test.

### 3.2 Summary of studies

Across LAC, the presence of the ε4 allele consistently showed strong associations with AD risk. In Argentina, ε4 was enriched in amyloid‐positive MCI^36^ and associated with AD (OR 3.3, 95% CI [1.2–9.0]).^37^ Similar associations were reported in Chile (2.9, [1.7–5.1]; frequency 20.6% and 40%),^51^, ^52^ Peru (5.02, [2.3–12.5]; frequency 9.2%),^67^ Ecuador (7.29, [2.82–18.80]; 2.01, [0.73–5.9]; frequency 33.9%),^58^, ^59^ Cuba (4.33, [1.27–14.79]; frequency 25%),^57^ Panama (5.14, [2.11–12.52]; frequency 57.1%),^65^, ^66^ with Jamaica reporting higher frequency in AD (39.2%).^60^ In Brazil, ε4 prevalence was higher in AD than in controls^38^, ^42^, ^43^, ^47^ and was significantly associated with AD, with ORs ranging from 2.43 [1.23–4.79] to 8.14 [4.57–14.49].^38^, ^41^, ^42^, ^43^, ^44^, ^48^, ^49^ In Colombia, ε4 carriers were more common in AD (66.0% vs. 25.6% in controls),^56^ with strong associations (7.6, [3.1–18.3]^53^ and 5.1, [1.9–13.6]^54^). Ancestry analyses showed a significant direct association with African ancestry (1.55, [1.09–2.03]) and an inverse association with Native American (0.75, [0.65–0.98]).^55^ In Venezuela, ε4 was linked to memory (2.29, [1.35–3.89]) and AD (1.52, [0.96–2.41] and (2.97, [1.87–4.71]).^69^, ^70^ In Mexico, ε4 was more frequent in dementia cases (22.4%),^63^ with strong association (13.33, [3.14–56.31]);^61^ and less common in Mexican Hispanics than non‐Hispanic Whites (21.4% vs. 42.9%);^62^ and *SORL1* interactions increased risk.^64^ In Brazil, ε3 showed OR < 1 estimates in allele‐count case–control comparisons (0.46, [0.30–0.67];^41^ 0.57, [0.30–1.06]^48^) and ε2 also showed OR < 1 in Brazil (0.16, [0.02–1.43])^48^ and Colombia (0.2, [0.05–0.75]).^54^ In Jamaica, ε2 had comparatively high frequency among AD cases (17.9%).^60^

The *APOE* ε4/ε4 genotype conferred the highest odds of AD. Strong associations were reported in Brazil (9.57, [2.07–44.13];^39^ 8.74, [2.83–27.02]^40^), Chile (12.8, [3.9‐47.6]),^52^ and Colombia (19.6, [2.3–161.9]).^53^ Ecuador showed weaker effects (2.15, [0.6–7.9]).^59^ In Venezuela, ε4/ε4 also shows high association (4.29, [1.32–13.90];^68^ 5.78, [1.24–26.85];^69^ 4.23, [1.17–15.29]), with specific effects in women (3.43, [2.04–5.76]) and older adults (10.02, [1.16–86.28]).^70^ The ε3/ε4 genotype shows moderate associations in Brazil (3.22, [1.73–6.01]),^39^ (3.63, [1.5–8.7]),^48^ and Venezuela (1.93, [1.016–3.69]) with ε3/ε3 showing lower odds in that study‐specific comparison (0.55, [0.307–1.014]).^68^

### 3.3 Frequency of *APOE* alleles and genotypes in the selected studies

According to the studies, *APOE* allele and genotype frequencies differed markedly between patients with AD versus controls. The ε2 allele (5.1% vs. 6.1%) and ε3 allele (67.3% vs. 82.6%) were less frequent in individuals with AD, whereas the ε4 allele was substantially higher in AD (27.7% vs. 11.3%). Genotype analysis showed slight increases in ε2/ε2 (0.8% vs. 0.6%) and ε2/ε4 (2.4% vs. 1.6%) among AD cases, along with a decrease in ε2/ε3 (6.1% vs. 9.6%). The ε3/ε3 genotype was notably reduced in AD (47.0% vs. 68.6%), while both ε3/ε4 (34.5% vs. 18.4%) and ε4/ε4 (9.3% vs. 1.3%) were substantially higher in patients with AD ∖ compared to controls[Table alz71224-tbl-0001] (Table 2).

**TABLE 2:** Meta‐analysis of the association between *APOE* alleles and AD.

	Numbers								
	Case		Control		Association test			Heterogeneity test	
	Events	Total	Events	Total	OR [95% CI]	*P*	Model	*P*	*I* ^2^ (%)
Allele									
ε2	324	6412	678	11,030	0.89 [0.76; 1.04]	0.1458	F	0.0034	46
ε3	4313	6412	9110	11,030	0.42 [0.37; 0.48]	<0.0001	R	<0.0001	54
ε4	1775	6412	1242	11,030	3.25 [2.82; 3.76]	<0.0001	R	0.0006	50
Genotype									
ε2/ε2	26	3206	31	5515	1.36 [0.81; 2.27]	0.2335	F	0.3757	7
ε2/ε3	196	3206	528	5515	0.60 [0.50; 0.72]	<0.0001	F	0.1380	21
ε2/ε4	76	3206	88	5515	1.35 [0.97; 1.87]	0.0709	F	0.1930	18
ε3/ε3	1506	3206	3785	5515	0.39 [0.35; 0.43]	<0.0001	F	0.0046	43
ε3/ε4	1105	3206	1012	5515	2.59 [2.31; 2.91]	<0.0001	F	0.0319	33
ε4/ε4	297	3206	71	5515	6.84 [5.09; 9.19]	<0.0001	F	0.9708	0

*Note*: The table summarizes the association between the *APOE* alleles and AD. It includes the number of cases and controls, the allele and genotype frequencies, the OR, and the corresponding CI. The table also reports the statistical significance for each analysis, with the model type indicated: F represents the fixed effect model, and R denotes the random effect model. Additionally, the table presents the heterogeneity test results, with *I* ^2^ values expressed as percentages.

Abbreviations: AD, Alzheimer's disease; *APOE*, apolipoprotein E; CI, confidence interval; OR, odds ratio.

### 3.4 Meta‐analysis results

#### 3.4.1 Association between the *APOE* allele and genotype and AD

Using allele‐count case–control comparisons (i.e., enrichment/depletion of each allele in AD cases vs. controls), we found that ε2 allele was not associated with AD (pooled OR 0.89, 95% CI [0.76–1.04], *P* = 0.146; Figure S1), whereas ε3 showed an OR < 1 in the allele‐count case–control comparison (0.42, [0.37–0.48], *P* < 0.0001; Figure 2). The presence of the ε4 allele showed a strong association with higher odds of AD (3.25, [2.82–3.76], *P* < 0.0001; Figure 3). To provide a consistent comparator aligned with common *APOE* epidemiologic practice, we additionally estimated ε2 and ε4 associations using ε3 as the reference/baseline allele. A significant indirect association was observed for the ε2 allele and AD (0.60, 95% CI [0.26–0.94], *P* < 0.0001; Figure S2 in supporting information), while ε4 was associated with AD (2.13, [1.85–2.42], *P* < 0.0001; Figure S3 in supporting information). Both analyses showed high heterogeneity (*I* ^2^ = 83% for ε2, 92% for ε4), reflecting variability across studies. When analyzed by *APOE* genotypes, ε3/ε4 and ε4/ε4 carriers had significantly higher odds of AD (2.59, [2.31–2.91]; 6.84, [5.09–9.19], both *P* < 0.0001), while ε2/ε3 showed OR < 1 relative to non‐ε2/ε3 genotypes in the pooled model. No significant associations were observed for ε2/ε2 or ε2/ε4 (Table 2). Given the disproportionate number of Brazilian studies (13 of 35 studies), we conducted sensitivity analyses excluding data from Brazil. Heterogeneity increased for the *APOE* ε2 allele, while it remained generally stable for the ε3 and ε4 alleles across models. Results remained directionally and statistically consistent (Figures S4–S6 in supporting information), supporting the stability of the pooled estimates.

#### 3.4.2 Country‐specific meta‐analysis of *APOE* alleles and genotypes

Country‐specific meta‐analyses of *APOE* alleles were performed for Argentina, Brazil, Chile, Colombia, Ecuador, Mexico, Panama, and Venezuela (Table S1 in supporting information). For ε2, significant effects were found only in Brazil, with lower odds (OR 0.74, 95% CI [0.58–0.95], *P* = 0.017), and in Mexico, with higher odds of AD (1.92, [1.34–2.74], *P* = 0.0003). The ε3 allele consistently showed ORs < 1 in all analyzed countries. For ε4, significant associations with AD were observed across all countries, with ORs from 2.72 in Chile ([1.88–3.96], *P* < 0.0001) to 4.48 in Mexico ([2.14–9.38], *P* < 0.0001).

Country genotype‐specific analyses (Table S2 in supporting information) showed very low ε2/ε2 and ε2/ε4 frequencies, preventing pooled estimates. The ε3/ε3 genotype was associated with lower AD odds in all countries, ranging from 0.29 in Ecuador ([0.20–0.42], *P* < 0.0001) to 0.61 in Panama ([0.45–0.83], *P* < 0.002). For ε2/ε3, significant lower associations with AD were found in Mexico (0.26, [0.14–0.49], *P* < 0.0001), Colombia (0.36, [0.20–0.65], *P* = 0.0007), and Brazil (0.57, [0.42–0.76], *P* < 0.0001). The ε3/ε4 genotype was associated with AD in most countries (except Chile and Venezuela), with ORs from 1.8 in Panama ([1.28–2.54], *P* = 0.0007) to 5.2 in Ecuador ([2.27–11.94], *P* < 0.0001). The ε4/ε4 genotype showed the strongest association with AD (except in Argentina), with ORs ranging from 3.22 in Panama ([1.24–8.42], *P* = 0.017) to 13.29 in Ecuador ([3.62–48.80], *P* < 0.0001).

### 3.5 Publication bias

Funnel plots of ε2 (Figure S7A in supporting information), ε3 (Figure S7B), and ε4 (Figure S7C) alleles indicated low publication bias. A Begg test^31^ confirmed no significant bias (ε2 *P*_begg_ = 0.114; ε3 *P*_begg_ = 0.402; and ε4 *P*_begg_ = 0.287).

## 4 DISCUSSION

This systematic review and meta‐analysis represent the first comprehensive synthesis to date of *APOE* allele and genotype associations with AD across 11 LAC countries (Argentina, Brazil, Chile, Colombia, Cuba, Ecuador, Jamaica, Mexico, Panama, Peru, and Venezuela), integrating evidence from 35 studies including 3206 AD cases and 5515 controls. Across analyses, ε4 emerged as the predominant genetic risk factor for AD in the region, while also revealing between‐country variability consistent with the complex interplay among genetic susceptibility, ancestry, and environmental exposures in shaping AD risk. Notably, the ε4/ε4 genotype exhibited the strongest association with AD, underscoring its relevance as a major risk factor in LAC populations. Interestingly, the ε3 allele exhibited a lower AD OR across analyses, consistent with its higher frequency among controls compared to AD cases while the impact of ε2 was heterogeneous across countries, highlighting contextual and study‐design sources of variability.

### 4.1 *APOE* physiopathology

apoE plays a central role in brain lipid transport, synaptic maintenance, and neuronal repair; however, its isoform‐specific properties critically shape vulnerability to AD. apoE2 and apoE3 generally support lipid redistribution and Aβ clearance, while apoE4 exhibits reduced stability and is more prone to proteolytic cleavage, producing neurotoxic fragments that disrupt mitochondrial function, impair cytoskeletal integrity, and promote tau hyperphosphorylation.^18^ In addition, apoE4 alters glutamatergic receptor trafficking and weakens blood–brain barrier integrity, amplifying neuroinflammation and synaptic dysfunction.^18^ These converging mechanisms provide a biological explanation for the strong association between *APOE* ε4 carriage and earlier onset, faster progression, and greater cognitive decline across the AD continuum.^71^ Recent neuropathological studies in Brazilian cohorts show that higher African ancestry is linked to fewer neuritic plaques and reduced ε4‐related AD risk, emphasizing ancestry's role in modulating *APOE* effects.^72^ The ε4 impact on cognition appears mainly mediated by AD neuropathology (neuritic plaques and neurofibrillary tangles), not by cerebrovascular or TAR DNA‐binding protein 43 lesions.^73^ These findings support that ancestry and neuropathological context shape *APOE*‐related risk in admixed LAC populations, consistent with the heterogeneity observed in our meta‐analysis.

### 4.2 Comparisons to global findings

The results of this meta‐analysis largely align with international evidence.^34^, ^35^, ^36^, ^37^, ^38^, ^39^, ^40^, ^41^, ^42^, ^43^, ^44^, ^45^, ^46^, ^47^, ^48^, ^49^, ^50^, ^51^, ^52^, ^53^, ^54^, ^55^, ^56^, ^57^, ^58^, ^59^, ^60^, ^61^, ^62^, ^63^, ^64^, ^65^, ^66^ Consistent with data from North America, Europe, Asia, and Africa,^11^, ^17^, ^74^, ^75^, ^76^, ^77^, ^78^ we observed a robust association between ε4 and AD, with carriers displaying > 3‐fold increase in odds relative to non‐carriers (Figure 3). In contrast, ε2 effects were heterogeneous. Comparing ε2 carriers versus non‐carriers, we did not observe a uniform effect, whereas ε2 showed an inverse association in ε3‐referenced models, suggesting that ε2‐related associations may be context dependent across populations and study designs.^76^, ^77^, ^78^, ^79^ We also observed consistently lower odds associated with ε3 across analyses. Given that ε3 is often treated as “neutral,” these results should be interpreted cautiously,^11^, ^74^, ^75^, ^76^, ^77^, ^78^, ^80^ as allele‐ and genotype‐frequency differences between cases and controls may reflect demographic structure, sampling processes, recruitment strategies, survival bias, population stratification, and contextual exposures rather than biological protection.

Although *APOE–*sex interactions have been described in other populations, with ε4 often conferring greater risk of AD in women, data from LAC remain extremely limited. Only three studies reported sex‐stratified associations, with heterogeneous designs and outcomes.^42^, ^69^, ^70^ While a formal meta‐analysis was not possible, descriptive trends suggest a potentially stronger ε4 effect in females.^17^, ^81^ Future studies in LAC populations should systematically report sex‐specific *APOE* effects to evaluate whether this interaction contributes to regional variability in AD risk.

Compared to global reference populations, the allele frequencies observed in our LAC meta‐analysis show both shared and distinctive features. ε4 frequency in LAC controls is comparable to European and North American cohorts^11^, ^20^, ^82^ and lower than in African‐derived populations,^83^, ^84^, ^85^ but its enrichment among AD cases is more pronounced,^11^, ^20^, ^75^, ^82^, ^86^, ^87^ while ε3 shows a sharper depletion in AD than generally reported elsewhere.^11^, ^20^, ^75^, ^82^, ^87^ Cross‐population comparisons should be interpreted cautiously due to differences in ancestry composition, ascertainment, and study design. Overall, the most robust finding is the marked case–control shift characterized by ε4 enrichment and ε3 depletion among AD cases, consistent with established ε4‐associated AD risk.

### 4.3 Regional and country‐specific insights

Our country‐specific analyses revealed important heterogeneity. Genotype‐based analyses confirmed that ε3/ε4 and especially ε4/ε4 are associated with substantially higher odds, with ε4/ε4 carriers exhibiting nearly 7‐fold higher odds.

While the ε4 allele conferred elevated risk in all countries, the magnitude varied considerably, with the highest effect observed in Mexico (OR 4.48) and the lowest in Brazil (OR 2.11). Ecuador exhibited an especially high OR for ε4/ε4 carriers (13.29), surpassing estimates from most Western populations and raising the possibility of region‐specific genetic or environmental modifiers amplifying risk.

The ε2 allele illustrated striking contrasts: it was associated with reduced AD risk in Brazil but was unexpectedly linked to higher odds in Mexico, highlighting that ε2 associations may vary across study contexts and populations. Such divergence may be driven by differences in genetic admixture (e.g., indigenous vs. European or African ancestry proportions), selective survival biases, or interaction with local environmental risk factors such as diet, cardiovascular burden, or access to health care.

The ε3 allele consistently demonstrated lower odds across nearly all LAC countries, indicating that its frequency distribution differs between AD cases and controls. However, given the exceptionally high AD prevalence in LAC,^4^, ^5^ it is plausible that social and environmental determinants, such as high prevalence of cardiovascular disease, socioeconomic disparities, and limited access to timely health care, shape these epidemiological patterns and influence how *APOE* associations manifest at the population level.^6^, ^88^, ^89^, ^90^, ^91^, ^92^, ^93^ In a Brazilian cohort, ε3 was more frequent among controls (77%) than patients with AD (60%), resulting in OR < 1 estimates for ε3 carriers (OR = 0.46), and for the ε3/ε3 genotype (OR = 0.36),^41^ compared to ε4. These findings reflected the case–control frequency differences; therefore, such differences should not be interpreted as evidence that ε3 exerts a biological protective effect.

Genotype‐specific analyses also confirm country‐level variability. The ε2/ε3 genotype showed lower odds only in Mexico, Colombia, and Brazil, whereas ε3/ε4 showed higher odds in most but not all countries (e.g., absent in Chile and Venezuela). These patterns may reflect admixture‐driven allele frequency differences, small sample sizes in underrepresented regions, or context‐specific interactions between *APOE* isoforms and modifiable risk factors. Although Brazil contributed a larger share of studies, the exclusion of Brazilian data yielded comparable pooled effects, suggesting that the overall findings are not driven by a single country. However, some residual heterogeneity may reflect country‐level clustering, which we partially addressed through random‐effects modeling and meta‐regression.

Recently, a meta‐analysis assessed AD risk among ε4 carriers in populations from Mexico, Panama, Cuba, Colombia, Venezuela, and Peru, and reported a pooled OR of 3.8, closely aligning with our finding (pooled OR 3.49); however, the association was only significant in the South American countries (pooled OR 4.61).^25^ Another meta‐analysis across different populations, including four studies from South America, using the e3 allele as a reference, reported a pooled OR of 2.45,^79^ similar to our finding (pooled OR 2.13). The minor discrepancy with our work could be attributed to the variability in the number of studies and participants included, population characteristics, and statistical approaches.

LAC countries exhibit some of the highest AD prevalence rates globally, yet the underlying factors remain largely unknown.^38^, ^39^, ^40^, ^41^, ^42^, ^43^, ^44^, ^45^, ^46^, ^47^, ^48^, ^49^, ^50^, ^51^, ^52^, ^53^, ^54^, ^55^, ^56^, ^57^, ^58^, ^59^, ^60^, ^61^, ^62^, ^63^, ^64^, ^65^, ^66^, ^67^, ^68^, ^69^, ^70^ Variations in *APOE* allele distributions across different populations may partially contribute to observed differences in risk estimates. The heterogeneous distribution of *APOE* isoforms in the LAC population might contribute to the significant disease burden observed. Previous meta‐analyses were limited to South America, included only a few studies from LAC, and primarily focused on the risk conferred by the ε4 allele.^26^, ^79^ This study is the first meta‐analysis included in a wide variety of LAC countries with a high number of studies and participants, evaluating the risk conferred by carriers versus non‐carriers of each allele and using ε3 allele as a reference and the genotypes.

When analyzed by *APOE* genotype, neither the ε2/ε2 nor the ε2/ε4 genotypes showed a significant association with AD in our study population. In contrast, the ε2/ε3 and especially the ε3/ε3 genotypes exhibited lower odds (pooled OR 0.60 and 0.39, respectively), underscoring that the OR < 1 observed in allele‐count analyses most plausibly reflects relative depletion of ε3 among cases driven by ε4 enrichment, rather than an intrinsic protective biological effect of ε3. These findings are consistent with Iranian, Indian, and Chinese cohorts, reporting no significant association for ε2/ε2 and ε2/ε4 and OR < 1 estimates for ε3/ε3 in case–control comparisons.^74^, ^75^, ^76^, ^77^, ^78^ Conversely, the ε3/ε4 and ε4/ε4 genotypes were strongly associated with an increased risk of AD (pooled OR 2.59 and 6.84, respectively), aligning with extensive literature identifying ε4 as a significant genetic risk factor across diverse populations.^11^, ^17^, ^74^, ^75^, ^76^, ^77^, ^78^, ^80^

Our country‐specific meta‐analysis confirms the critical role of *APOE* alleles in AD risk within LAC countries and reveals significant variations across countries. The ε2 allele, often associated with lower odds of AD relative to ε3 in many non‐LAC studies, showed contrasting results in our study. In Brazil, ε2 was linked to a significantly reduced odds of AD (OR 0.74), whereas in Mexico, it was unexpectedly associated with higher odds of AD (OR 1.92). This discrepancy may be influenced by genetic admixture, sample composition, or environmental interactions.^88^, ^89^, ^94^, ^95^ The ε3 allele was consistently associated with lower AD odds across all LAC countries analyzed. However, given the high burden of AD in LAC countries, factors such as high cardiovascular disease prevalence, socioeconomic disparities, and other environmental factors^6^, ^88^, ^89^, ^90^, ^91^, ^92^, ^93^ may modify the observed case–control frequency patterns and effect estimates, without implying that ε3 exerts a protective biological effect. In our meta‐analysis, the ε4 allele emerges as the primary genetic risk factor for AD, with significant associations observed in all analyzed countries, aligning with findings from other global populations. However, the strength of the association varied by country, with Mexico showing the higher odds (OR 4.48). This heterogeneity may reflect differences in sample sizes, genetic backgrounds, or environmental exposures. Our country‐specific genotype‐based meta‐analysis further supports these allele‐specific trends. The ε3/ε3 genotype was consistently associated with a lower odds of AD, with ORs ranging from 0.29 in Ecuador to 0.61 in Panama. The ε2/ε3 genotype showed lower odds only in Mexico, Colombia, and Brazil, aligning with global findings in which ε2 carriers show reduced susceptibility. The ε3/ε4 genotype, representing an intermediate‐risk profile, was significantly associated with AD in most countries except Chile and Venezuela, suggesting potential genetic or environmental modifiers affecting its impact. Notably, the ε4/ε4 genotype showed the strongest association with AD across LAC, consistent with global evidence of its substantial risk contribution. Notably, Ecuador had the highest OR (13.29), exceeding estimates from Western populations, highlighting possible regional differences in genetic risk expression.

Genotype distributions reinforced the allele‐level patterns. The ε3/ε3 genotype was highly prevalent among LAC controls (68.6%) but showed a substantial reduction in AD cases (46.9%), representing a sharper decline than typically reported in non‐LAC populations.^11^, ^20^, ^82^, ^86^ In contrast, ε3/ε4 and ε4/ε4 genotypes were markedly overrepresented among AD patients.^11^, ^20^, ^75^, ^82^, ^86^ These patterns suggest that variation in observed effect sizes across LAC is primarily driven by differences in genotype frequencies, ancestry composition, sampling frames, and study‐design heterogeneity, rather than population‐specific differences in apoE isoform biology.

### 4.4 Strengths and limitations of the study

The primary strength of this meta‐analysis lies in its scope and representativeness. It is the first to include a large, diverse set of LAC countries, substantially expanding beyond prior studies restricted to South America or limited subsets of data.^26^, ^80^ By pooling results from 3206 AD cases and 5515 controls across 11 countries, we were able to derive more precise estimates of allele‐ and genotype‐specific odds, and to explore regional variability with greater confidence. Another strength is the systematic evaluation of all major apoE isoforms and genotypes. Unlike earlier analyses that focused predominantly on ε4, our study examined alleles associated with higher odds and inverse association, including explicit evaluation of ε2‐ and ε3‐related frequency patterns. By using both carriers versus non‐carriers and ε3‐referenced models, we provide a more nuanced assessment of allele effects.

However, certain limitations must be acknowledged. Although this analysis included 11 countries, representation was uneven, with Brazil contributing most cases and several countries represented by only one or two studies. As a result, the pooled effect sizes may disproportionately reflect countries with larger samples, while underrepresenting smaller or less‐studied populations. Variability in study designs, sample sizes, and diagnostic criteria across included studies may introduce heterogeneity, influencing the reliability of pooled estimates. Efforts to obtain additional allele frequency and genotype data were largely unsuccessful, with data received via e‐mail in only three cases. Additionally, potential confounding factors such as ancestry and population stratification were not addressed, which could influence the interpretation of results. Within‐country variability in ancestry composition (e.g., greater Amerindian background in northern Brazil and Mexico vs. predominantly European ancestry in southern Brazil and Argentina) may influence *APOE* effect sizes. However, because ancestry data were seldom reported, we could not adjust for this factor. Future LAC studies integrating genomic ancestry estimates are needed to clarify whether local ancestry modifies *APOE*‐associated AD risk. Consequently, our findings should be interpreted as genetic associations rather than comprehensive risk models.

In summary, our findings indicate that the *APOE* ε4 allele significantly increases AD odds in LAC populations. Environmental, socioeconomic, and other modifiable factors may attenuate the risk of *APOE*, particularly in regions with high dementia prevalence.^38^, ^39^, ^40^, ^41^, ^42^, ^43^, ^44^, ^45^, ^46^, ^47^, ^48^, ^49^, ^50^, ^51^, ^52^, ^53^, ^54^, ^55^, ^56^, ^57^, ^58^, ^59^, ^60^, ^61^, ^62^, ^63^, ^64^, ^65^, ^66^, ^67^, ^68^, ^69^, ^70^ The variability in the impact of *APOE* alleles across countries underscores the complex interplay among genetic, environmental, and social determinants in shaping AD risk.^6^, ^93^ Future research in well‐characterized cohorts, such as those within the Multi‐Partner Consortium to Expand Dementia Research in Latin America (ReDLat),^96^ will be essential to disentangle these interactions. Integrating social determinants of health into these studies, which have been shown to significantly influence AD, will provide a more comprehensive framework for understanding AD in this region.

## CONFLICT OF INTEREST STATEMENT

The authors declare no conflicts of interest. Author disclosures are available in the supporting information.

## Supporting information



Supporting Information

Supporting Information
