# Supplementary material for: Association of apolipoprotein E variants on Alzheimer's disease in Latin America: A systematic review and meta‐analysis
Source: Alzheimers Dement. 2026 Feb 22;22(2):e71224. doi: 10.1002/alz.71224 (PMC12927995; doi:10.1002/alz.71224)
Supplement: Supplementary file 2 — Supporting Information [file ALZ-22-e71224-s001.docx]

**SUPPLEMENTARY FILES**


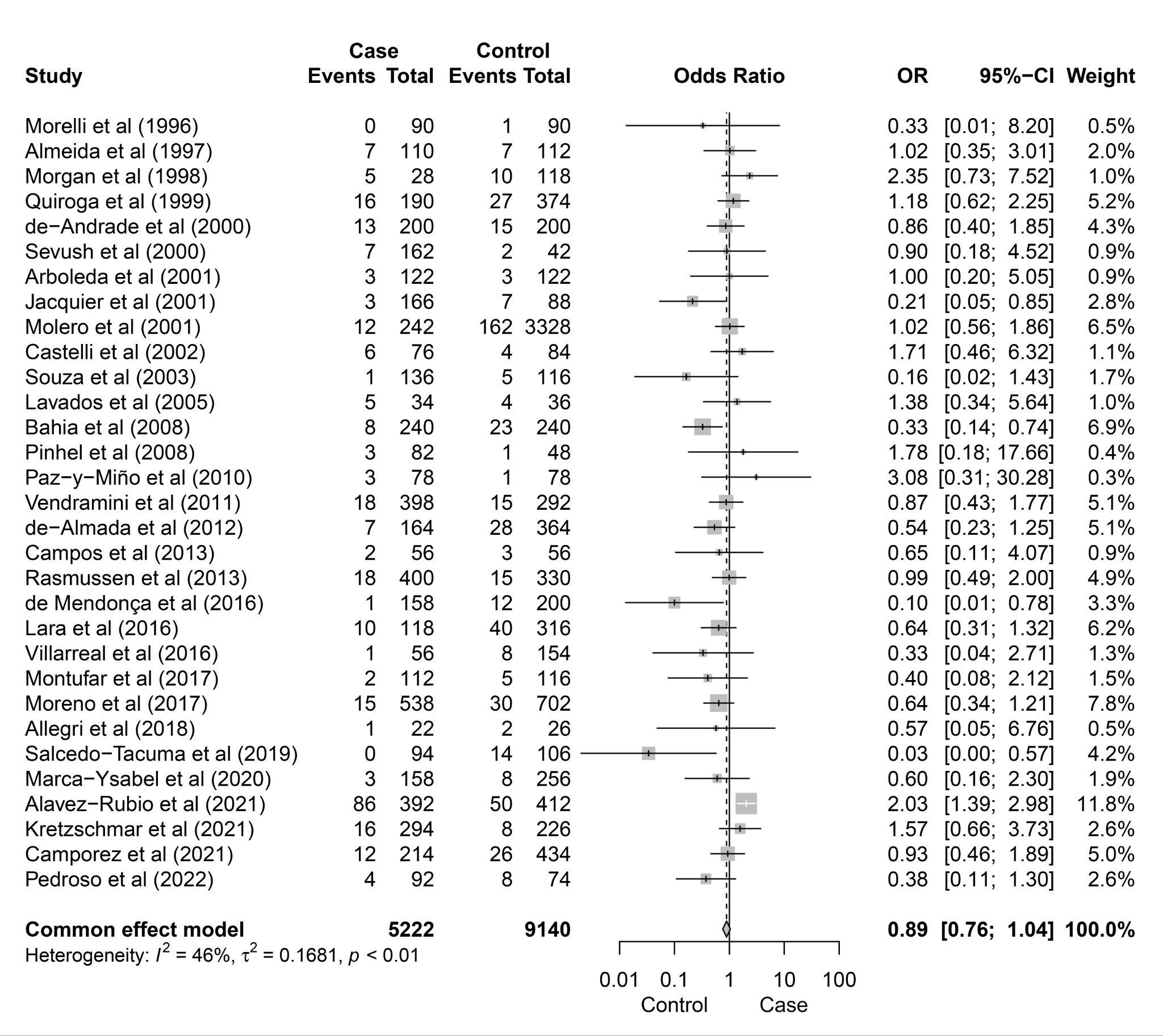


**Figure S1. Forest plot displaying the results of a meta-analysis evaluating the association between the *ApoE* ε2 allele and AD across 31 studies.** Each study's OR and 95% confidence interval (CI) are shown, with square markers representing individual study estimates, sized according to their relative weight in the meta-analysis. The pooled OR under the fixed-effect model is 0.89 (95% CI: 0.76–1.04), indicating a non-significant effect of *ApoE* ε2 against AD. Moderate heterogeneity was observed (I2=46%), suggesting some study variability. The overall results are visually summarized with the diamond at the bottom of the plot, reflecting the combined effect estimate and CI. OR =odd ratio; τ^2^ =between-study variance; p =p value.


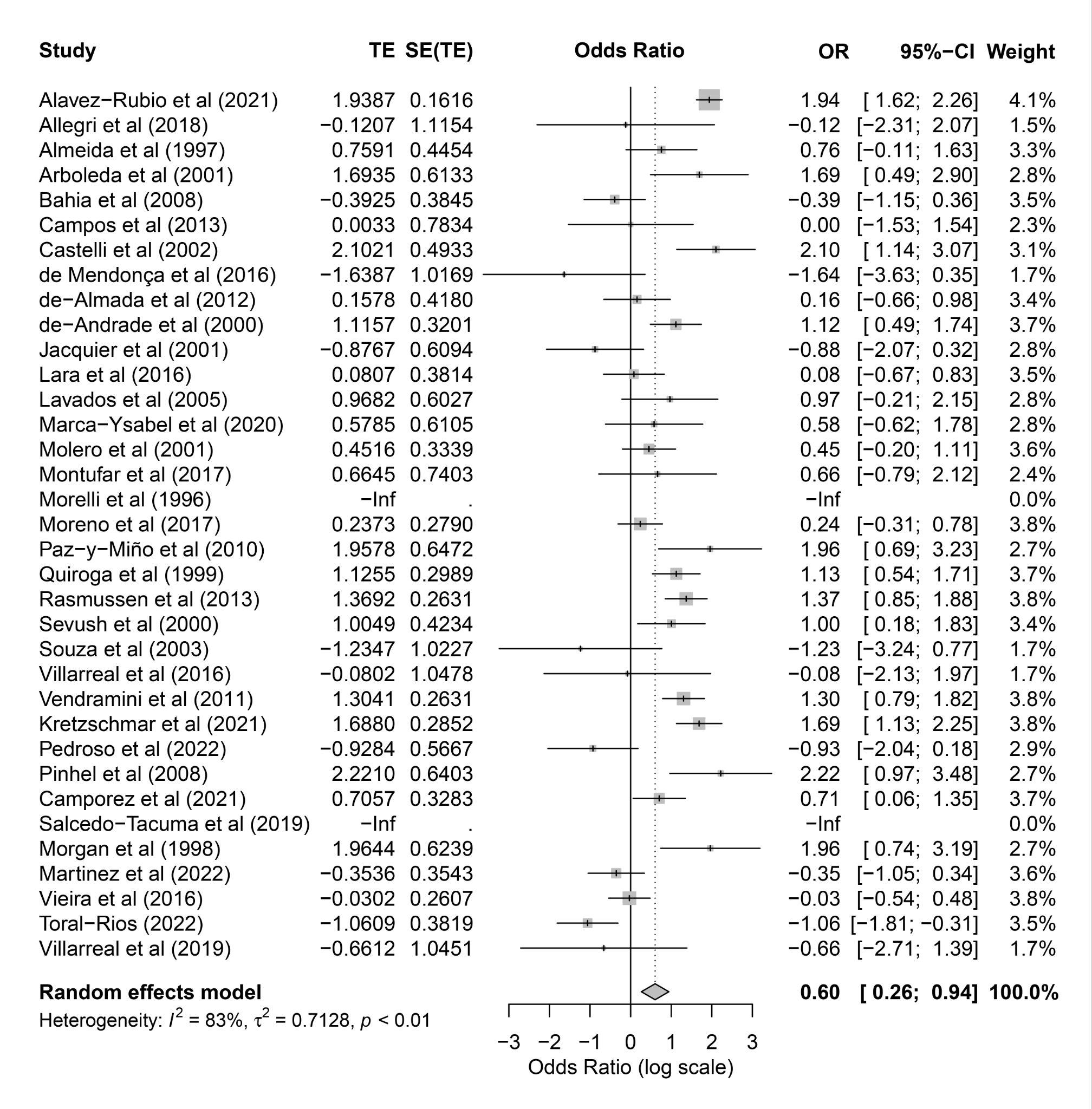


**Figure S2.** **Forest plot displaying the results of a meta-analysis evaluating the association between the *ApoE* ε2 allele and AD, using *ApoE* ε3 allele as reference, across 35 studies.** Each study's OR and 95% confidence interval (CI) are shown, with square markers representing individual study estimates, sized according to their relative weight in the meta-analysis. The pooled OR calculated using a random-effect model, is 0.60 (95% CI [ 0.26–0.94]), indicating a significant protective effect of *ApoE* ε2 against AD. High heterogeneity was observed (I2=83%), suggesting some study variability. The overall results are visually summarized with the diamond at the bottom of the plot, reflecting the combined effect estimate and CI. OR =odd ratio; τ^2^ =between-study variance; p =p value.


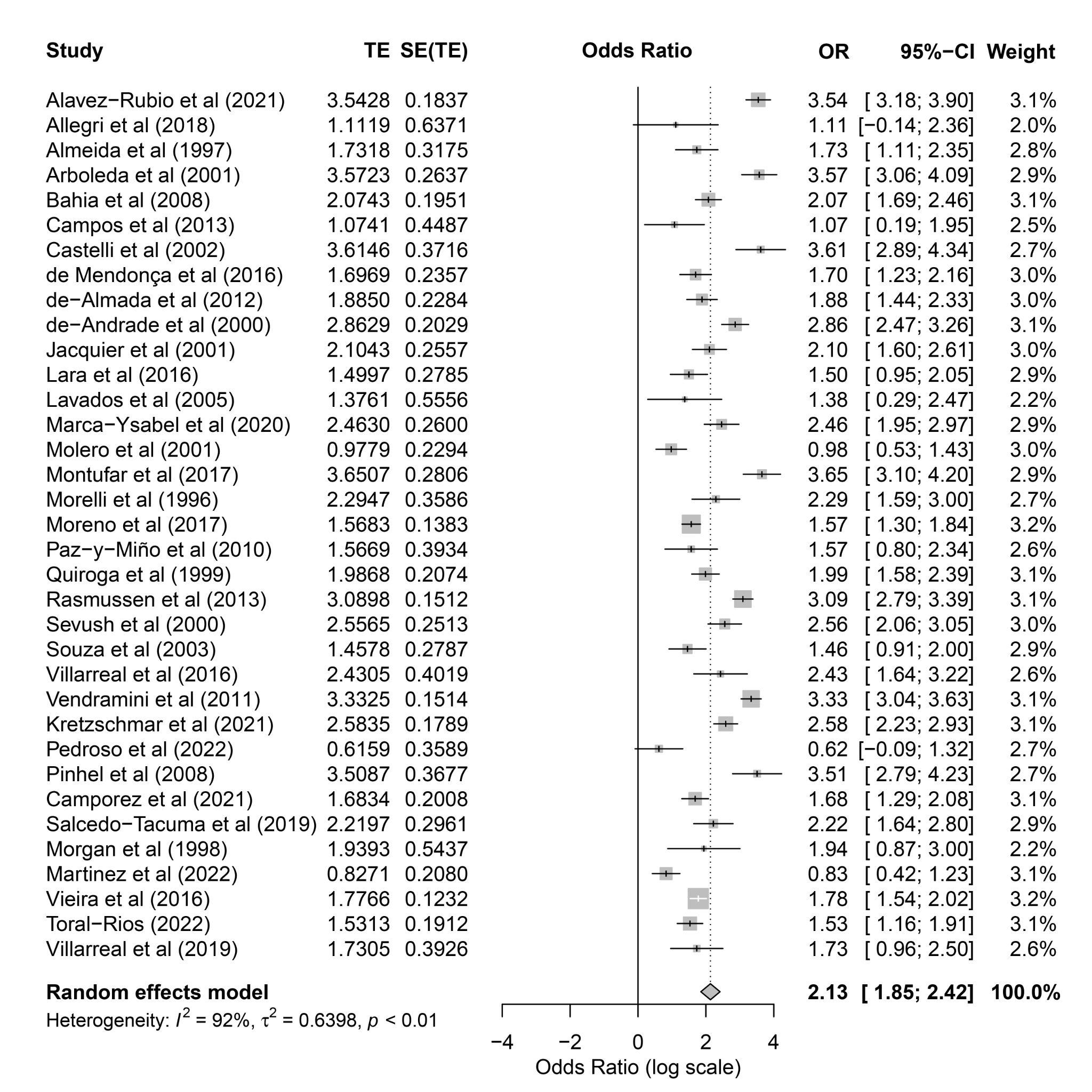


**Figure S3.** **Forest plot displaying the results of a meta-analysis evaluating the association between the *ApoE* ε4 allele and AD, using *ApoE* ε3 allele as reference, across 35 studies.** Each study's OR and 95% confidence interval (CI) are shown, with square markers representing individual study estimates, sized according to their relative weight in the meta-analysis. The pooled OR, calculated using a random-effect model, is 2.13 (95% CI [1.85–2.42], p =0.001), indicating a significant association between the *ApoE* ε4 allele and increased risk of AD. High heterogeneity was observed (I2=92%), indicating considerable variability across the included studies. The overall results are visually summarized with the diamond at the bottom of the plot, reflecting the combined effect estimate and CI. OR =odd ratio; τ^2^ =between-study variance; p =p value.


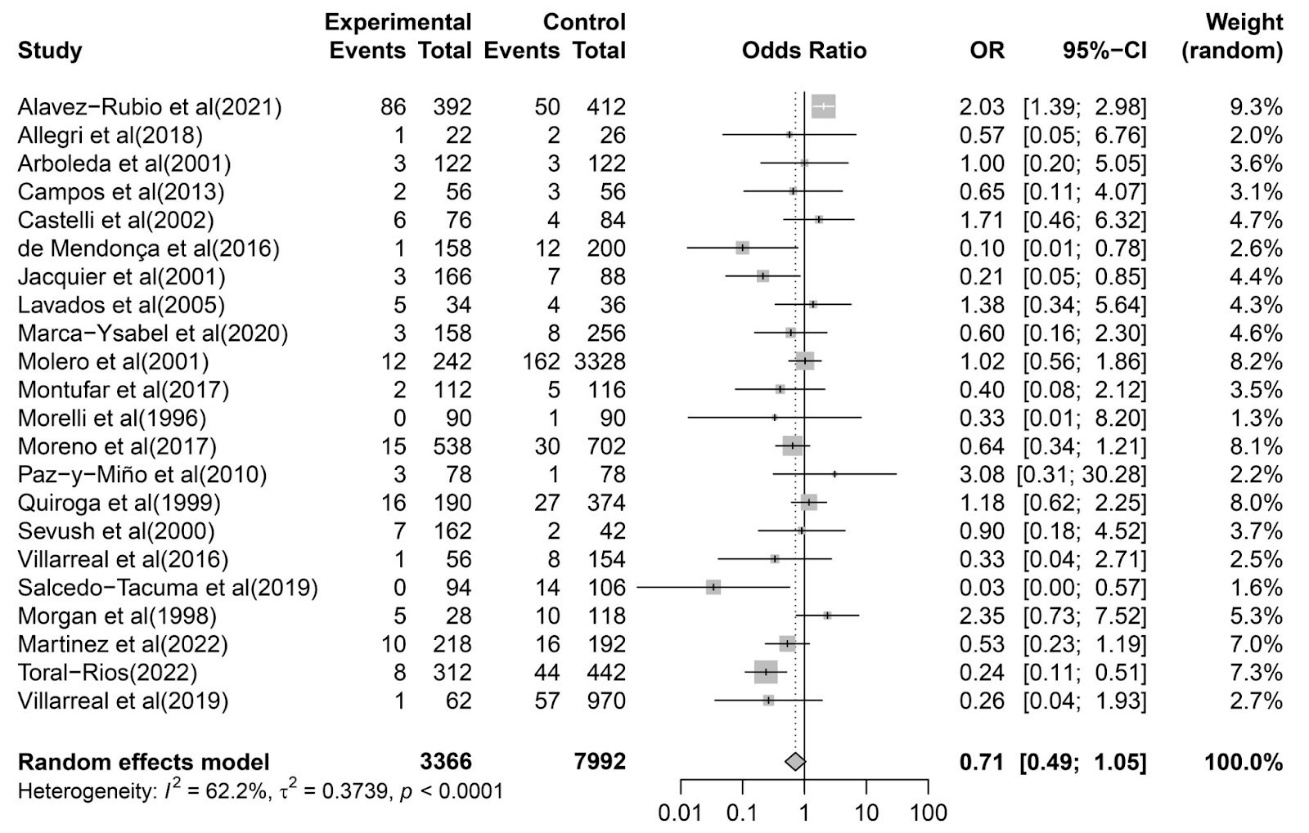


**Figure S4. Forest plot displaying the results of a meta-analysis evaluating the association between the *ApoE* ε2 allele and AD excluding Brazilian studies.** Each study's OR and 95% confidence interval (CI) are shown, with square markers representing individual study estimates, sized according to their relative weight in the meta-analysis. The pooled OR under the random-effect model is 0.71 (95% CI: 0.49–1.05), indicating a non-significant effect of *ApoE* ε2 against AD. High heterogeneity was observed (I2=62.2%), indicating considerable variability across the included studies. The overall results are visually summarized with the diamond at the bottom of the plot, reflecting the combined effect estimate and CI. OR =odd ratio; τ^2^ =between-study variance; p =p value.


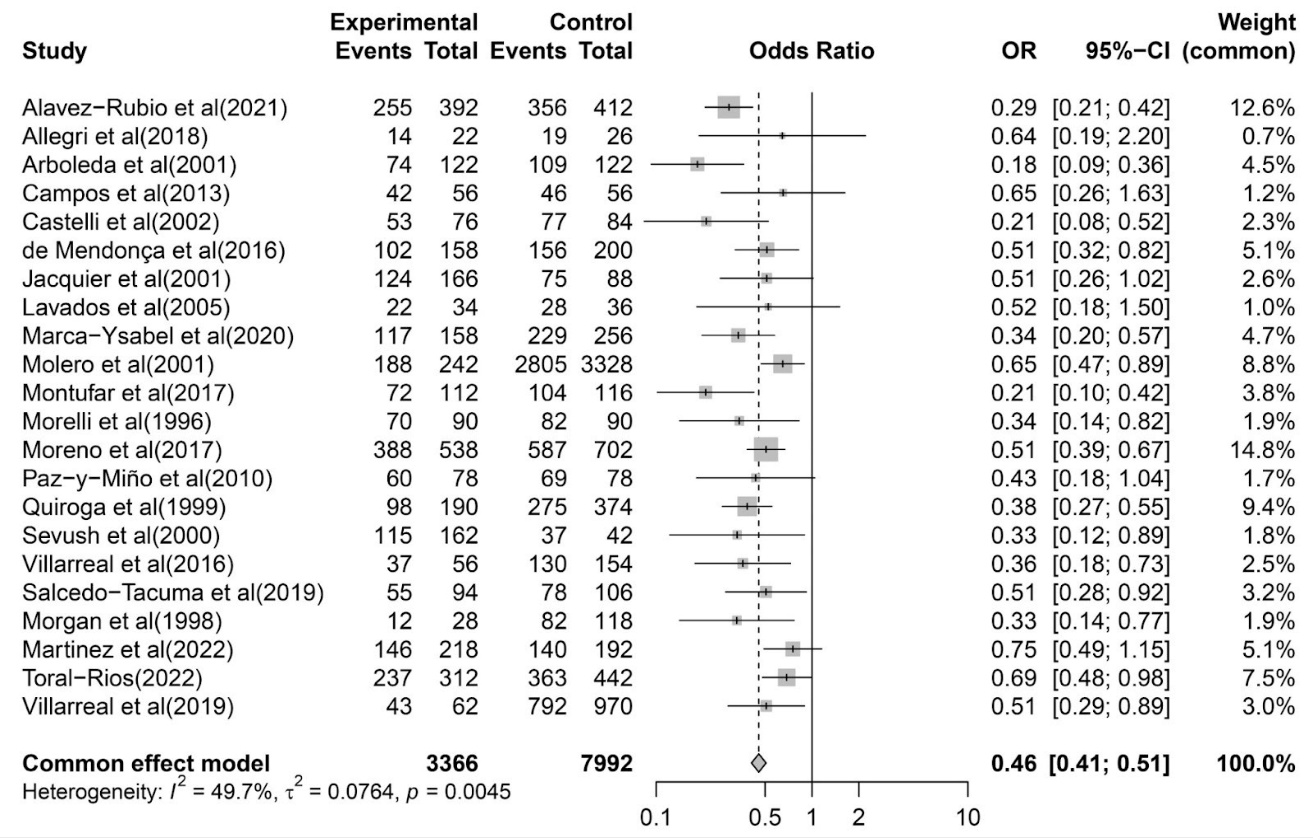


**Figure S5. Forest plot displaying the results of a meta-analysis evaluating the association between the *ApoE* ε3 allele and AD excluding Brazilian studies.** Each study's OR and 95% confidence interval (CI) are shown, with square markers representing individual study estimates, sized according to their relative weight in the meta-analysis. The pooled OR under the random-effect model is 0.46 (95% CI: 0.41–0.51), indicating a significant protective effect of *ApoE* ε3 against AD. Moderate heterogeneity was observed (I2=49.7%), indicating some variability across the included studies. The overall results are visually summarized with the diamond at the bottom of the plot, reflecting the combined effect estimate and CI. OR =odd ratio; τ^2^ =between-study variance; p =p value.


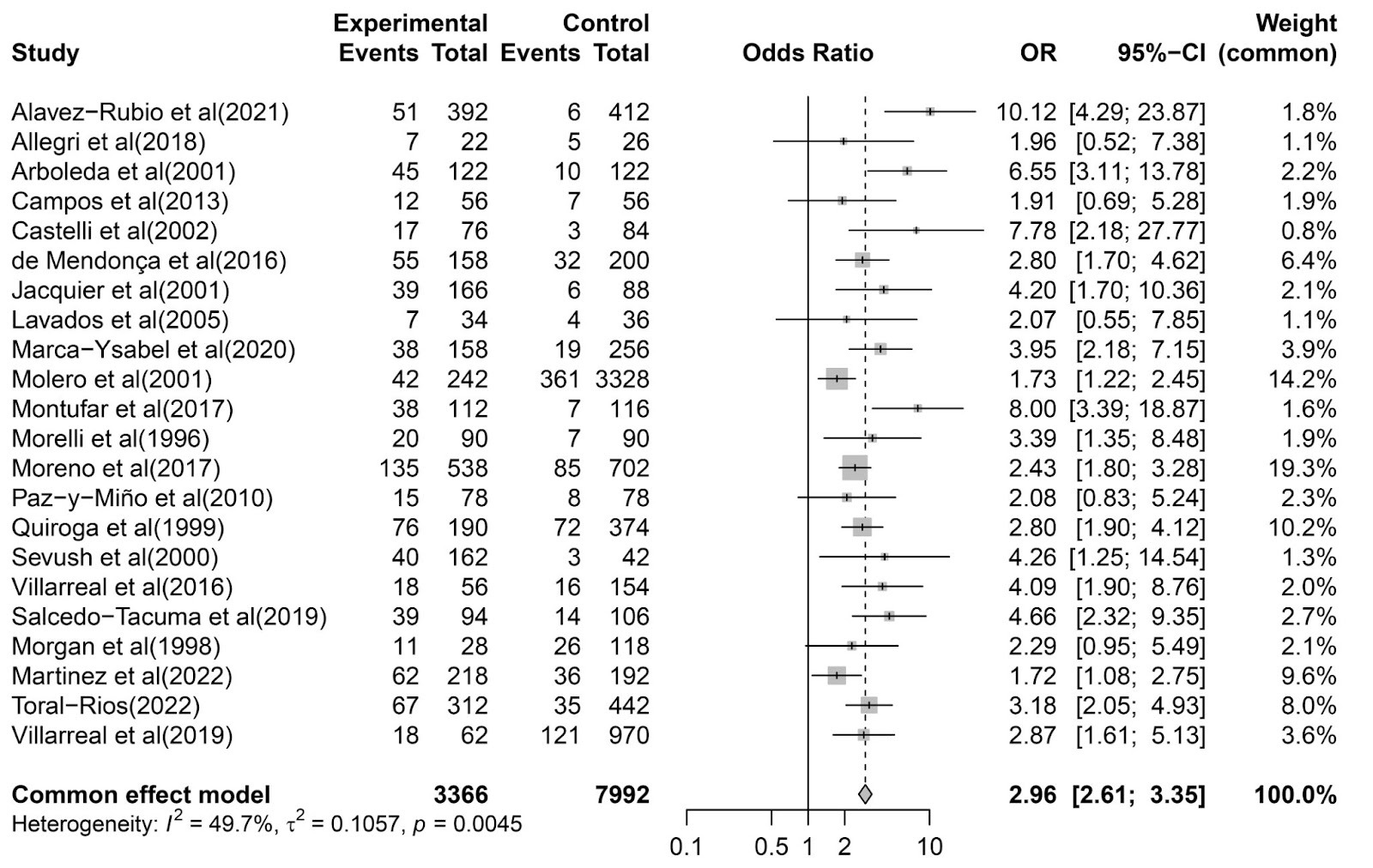


**Figure S6. Forest plot displaying the results of a meta-analysis evaluating the association between the *ApoE* ε4 allele and AD excluding Brazilian studies.** Each study's OR and 95% confidence interval (CI) are shown, with square markers representing individual study estimates, sized according to their relative weight in the meta-analysis. The pooled OR under the random-effect model is 2.96 (95% CI: 2.61–3.35), indicating a significant association between the *ApoE* ε4 allele and increased risk of AD. Moderate heterogeneity was observed (I2=49.7%), indicating some variability across the included studies. The overall results are visually summarized with the diamond at the bottom of the plot, reflecting the combined effect estimate and CI. OR =odd ratio; τ^2^ =between-study variance; p =p value.


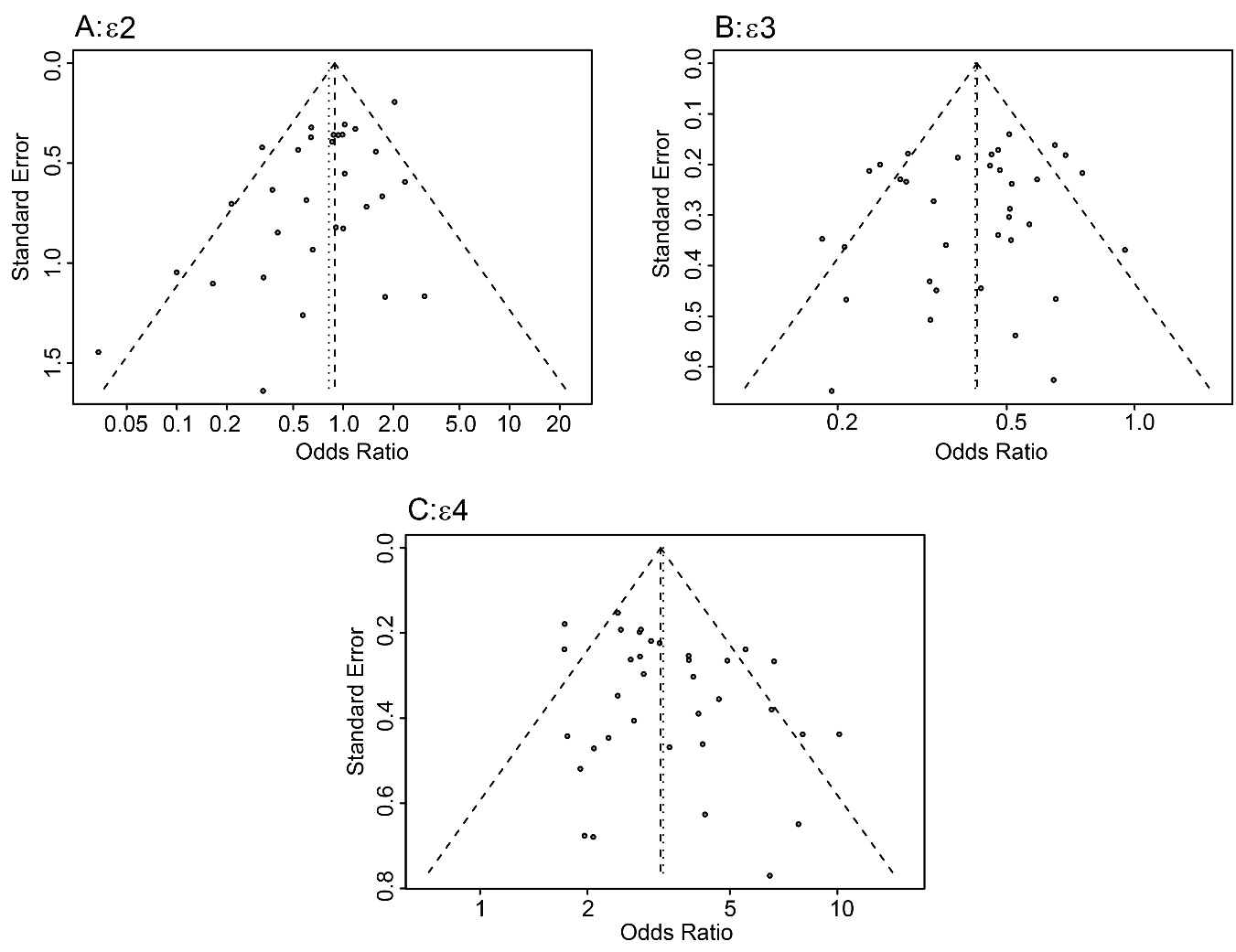


**Figure S7.** Funnel plots on the association between *ApoE* alleles and AD. **A.** Allele ε2, **B.** Allele ε3, **C.** Allele ε4. They show no obvious publication bias.

**Table S1. Meta-analysis of the association between *ApoE* alleles and AD by country.** The table summarizes the association between the *ApoE* alleles and AD by country. It includes the number of studies, number of cases and controls, the Odds Ratio (OR), and the corresponding Confidence Interval (CI). The table also reports the statistical significance for each analysis, with the model type indicated: F represents the fixed effect model and R denotes the random effect model. Additionally, the table presents the results of the heterogeneity test, with I² values expressed as percentages. OR=odd ratio; p =p value, * =crude.

|  |  | **Numbers** | | | | | **Association test** | |  | **Heterogeneity test** | |
| --- | --- | --- | --- | --- | --- | --- | --- | --- | --- | --- | --- |
| **Allele** | **Country** | **Studies**  **Number** | **Case** | | **Control** | | **OR [95% CI]** | **p** | **Model** | **p** | **I2 (%)** |
|  |  |  | **Event** | **Total** | **Event** | **Total** |  |  |  |  |  |
|  | Argentina | 2 | 1 | 112 | 3 | 116 | 0.46 [0.07; 3.22] | 0.4345 | F | 0.7903 | 0 |
|  | Brazil | 13 | 134 | 3046 | 208 | 3038 | 0.74 [0.58; 0.95] | **0.0168** | F | 0.3124 | 14 |
|  | Chile | 2 | 21 | 224 | 31 | 410 | 1.21 [0.68; 2.18] | 0.5149 | F | 0.8449 | 0 |
|  | Colombia | 4 | 21 | 920 | 54 | 1018 | 0.37 [0.11; 1.23] | 0.1045 | R | 0.1002 | 52 |
|  | Cuba | 1 | 7 | 162 | 2 | 42 | 0.90 [0.18; 4.52] | 0.9014 | * |  |  |
| **ε2** | Ecuador | 2 | 5 | 190 | 6 | 194 | 0.95 [0.13; 6.81] | 0.9611 | R | 0.1586 | 50 |
|  | Jamaica | 1 | 5 | 28 | 10 | 118 | 2.35 [0.73; 7.52] | 0.1507 | * |  |  |
|  | Mexico | 4 | 102 | 836 | 101 | 994 | 1.92 [1.34; 2.74] | **0.0003** | F | 0.4845 | 0 |
|  | Panama | 2 | 2 | 118 | 65 | 1124 | 0.33 [0.04; 2.71] | 0.3036 | F |  |  |
|  | Peru | 1 | 3 | 158 | 8 | 256 | 0.60 [0.16; 2.30] | 0.4556 | * |  |  |
|  | Venezuela | 3 | 23 | 618 | 190 | 3720 | 0.40 [0.04; 3.71] | 0.4168 | R | 0.0331 | 78 |
|  | Argentina | 2 | 84 | 112 | 101 | 116 | 0.42 [0.21; 0.85] | 0.0162 | R | 0.4093 | 0 |
|  | Brazil | 13 | 1989 | 3046 | 2417 | 3038 | 0.40 [0.33; 0.50] | <0.0001 | F | 0.0033 | 59 |
|  | Chile | 2 | 120 | 224 | 303 | 410 | 0.40 [0.28; 0.56] | <0.0001 | R | 0.5840 | 0 |
|  | Colombia | 4 | 641 | 920 | 849 | 1018 | 0.41 [0.26; 0.66] | 0.0002 | F | 0.0540 | 61 |
|  | Cuba | 1 | 115 | 162 | 37 | 42 | 0.33 [0.12; 0.89] | 0.0290 | * |  |  |
| **ε3** | Ecuador | 2 | 132 | 190 | 173 | 194 | 0.28 [0.14; 0.59] | 0.0007 | F | 0.1982 | 40 |
|  | Jamaica | 1 | 12 | 28 | 82 | 118 | 0.33 [0.14; 0.77] | 0.0100 | * |  |  |
|  | Mexico | 4 | 587 | 836 | 842 | 994 | 0.42 [0.24; 0.73] | 0.0021 | R | 0.0023 | 79 |
|  | Panama | 2 | 80 | 118 | 922 | 1124 | 0.44 [0.29; 0.68] | 0.0003 | F | 0.4509 | 0 |
|  | Peru | 1 | 117 | 158 | 229 | 256 | 0.34 [0.20; 0.57] | <0.0001 | * |  |  |
|  | Venezuela | 3 | 436 | 618 | 3101 | 3720 | 0.64 [0.51; 0.80] | <0.0001 | F | 0.4915 | 0 |
|  | Argentina | 2 | 27 | 112 | 12 | 116 | 2.86 [1.36; 6.06] | 0.0058 | F | 0.5058 | 0 |
|  | Brazil | 13 | 923 | 3046 | 359 | 3038 | 3.45 [2.81; 4.22] | <0.0001 | F | 0.0378 | 45 |
|  | Chile | 2 | 83 | 224 | 76 | 410 | 2.72 [1.88; 3.96] | <0.0001 | F | 0.6727 | 0 |
|  | Colombia | 4 | 258 | 920 | 115 | 1018 | 3.77 [2.42; 5.88] | <0.0001 | R | 0.0430 | 63 |
|  | Cuba | 1 | 40 | 162 | 3 | 42 | 4.26 [1.25; 14.5] | 0.0206 | * |  |  |
| **ε4** | Ecuador | 2 | 53 | 190 | 15 | 194 | 4.13 [1.10; 15.4] | 0.0350 | R | 0.0365 | 77 |
|  | Jamaica | 1 | 11 | 28 | 26 | 118 | 2.29 [0.96; 5.49] | 0.0634 | * |  |  |
|  | Mexico | 4 | 147 | 836 | 51 | 994 | 4.48 [2.14; 9.38] | <0.0001 | R | 0.0351 | 65 |
|  | Panama | 2 | 36 | 118 | 137 | 1124 | 3.31 [2.09; 5.23] | <0.0001 | R | 0.4706 | 0 |
|  | Peru | 1 | 38 | 158 | 19 | 256 | 3.95 [2.18; 7.14] | <0.0001 | * |  |  |
|  | Venezuela | 3 | 159 | 618 | 429 | 3720 | 1.95 [1.53; 2.29] | <0.0001 | F | 0.2516 | 28 |

**Table S2. Meta-analysis of the association between *ApoE* genotypes and AD by country.** The table summarizes the association between the *ApoE* genotypes and AD by country. It includes the number of studies, the number of cases and controls, the Odds Ratio (OR), and the corresponding Confidence Interval (CI). The table also reports the statistical significance for each analysis, with the model type indicated: F represents the fixed effect model and R denotes the random effect model. Additionally, the table presents the heterogeneity test results, with I² values expressed as percentages. OR=odd ratio; p =p value, * =crude.

| **Argentina** | **Numbers** | | | | **Association test** | |  | **Heterogeneity test** | |
| --- | --- | --- | --- | --- | --- | --- | --- | --- | --- |
| **Genotypes** | **Case** | | **Control** | | **OR[95% CI]** | **p** | **Model** | **p** | **I2 (%)** |
|  | **Events** | **Total** | **Events** | **Total** |  |  |  |  |  |
| ε2/ε2 | 0 | 56 | 0 | 58 | NA |  |  |  |  |
| ε2/ε3 | 1 | 56 | 3 | 58 | 0.44 [0.06; 3.24] | 0.4239 | F | 0.8032 | 0 |
| ε2/ε4 | 0 | 56 | 0 | 58 | NA |  |  |  |  |
| ε3/ε3 | 32 | 56 | 43 | 58 | 0.42 [0.18; 0.96] | 0.0402 | F | 0.5198 | 0 |
| ε3/ε4 | 19 | 56 | 12 | 58 | 2.07 [0.87; 4.90] | 0.0978 | F | 0.5352 | 0 |
| ε4/ε4 | 4 | 56 | 0 | 58 | 5.80 [0.64; 52.28] | 0.1173 | F | 0.7701 | 0 |
|  |  |  |  |  |  |  |  |  |  |
| **Brazil** | **Numbers** | | | | **Association test** | |  | **Heterogeneity Test** | |
| **Genotypes** | **Case** | | **Control** | | **OR[95% CI]** | **p** | **Model** | **p** | **I2 (%)** |
|  | **Events** | **Total** | **Events** | **Total** |  |  |  |  |  |
| ε2/ε2 | 5 | 1523 | 15 | 1519 | 0.55 [0.24; 1.24] | 0.1484 | F | 0.8122 | 0 |
| ε2/ε3 | 78 | 1523 | 144 | 1519 | 0.57 [0.42; 0.76] | 0.0001 | F | 0.3732 | 7 |
| ε2/ε4 | 46 | 1523 | 34 | 1519 | 1.54 [0.97; 2.45] | 0.0695 | F | 0.4424 | 0.3 |
| ε3/ε3 | 674 | 1523 | 1021 | 1519 | 0.36 [0.28; 0.45] | < 0.0001 | R | 0.0086 | 55 |
| ε3/ε4 | 563 | 1523 | 285 | 1519 | 2.73 [2.29; 3.25] | < 0.0001 | F | 0.0588 | 41 |
| ε4/ε4 | 157 | 1523 | 20 | 1519 | 8.18 [5.10; 13.11] | < 0.0001 | F | 0.9219 | 0 |
| **Panamá** | **Numbers** | | | | **Association test** | |  | **Heterogeneity test** | |
| **Genotypes** | **Case** | | **Control** | | **OR[95% CI]** | **p** | **Model** | **p** | **I2 (%)** |
|  | **Events** | **Total** | **Events** | **Total** |  |  |  |  |  |
| ε2/ε2 | 0 | 59 | 4 | 562 | 1.24 [0.07; 22.59] | 0.8837 | F |  | NA |
| ε2/ε3 | 2 | 59 | 47 | 562 | 0.94 [0.52; 1.71] | 0.8437 | F | 0.2203 | 33 |
| ε2/ε4 | 0 | 59 | 10 | 562 | 0.64 [0.26; 1.58] | 0.3300 | F | 0.7926 | 0 |
| ε3/ε3 | 25 | 59 | 375 | 562 | 0.61 [0.45; 0.83] | 0.0019 | F | 0.5776 | 0 |
| ε3/ε4 | 28 | 59 | 125 | 562 | 1.80 [1.28; 2.54] | 0.0007 | F | 0.7986 | 0 |
| ε4/ε4 | 4 | 59 | 1 | 562 | 3.22 [1.24; 8.42] | 0.0168 | F | 0.2470 | 25 |
| **Chile** | **Numbers** | | | | **Association test** | |  | **Heterogeneity test** | |
| **Genotypes** | **Case** | | **Control** | | **OR[95% CI]** | **p** | **Model** | **p** | **I2 (%)** |
|  | **Events** | **Total** | **Events** | **Total** |  |  |  |  |  |
| ε2/ε2 | 2 | 112 | 1 | 205 | 4.00 [0.36; 44.68] | 0.2602 | F |  | NA |
| ε2/ε3 | 11 | 112 | 29 | 205 | 0.64 [0.31; 1.34] | 0.2356 | F | 0.7162 | 0 |
| ε2/ε4 | 6 | 112 | 0 | 205 | 11.32 [1.35; 94.72] | 0.0252 | F | 0.8311 | 0 |
| ε3/ε3 | 33 | 112 | 104 | 205 | 0.39 [0.24; 0.64] | 0.0002 | F | 0.3398 | 0 |
| ε3/ε4 | 43 | 112 | 66 | 205 | 1.37 [0.84; 2.22] | 0.2045 | F | 0.7539 | 0 |
| ε4/ε4 | 17 | 112 | 5 | 205 | 7.93 [2.83; 22.26] | < 0.0001 | F |  | NA |
| **Colombia** | **Numbers** | | | | **Association test** | |  | **Heterogeneity test** | |
| **Genotypes** | **Case** | | **Control** | | **OR[95% CI]** | **p** | **Model** | **p** | **I2 (%)** |
|  | **Events** | **Total** | **Events** | **Total** |  |  |  |  |  |
| ε2/ε2 | 0 | 460 | 0 | 509 | NA |  |  |  |  |
| ε2/ε3 | 15 | 460 | 44 | 509 | 0.36 [0.20; 0.65] | 0.0007 | F | 0.2546 | 26 |
| ε2/ε4 | 6 | 460 | 10 | 509 | 0.72 [0.28; 1.81] | 0.4829 | F | 0.3250 | 14 |
| ε3/ε3 | 224 | 460 | 358 | 509 | 0.39 [0.29; 0.50] | < 0.0001 | F | 0.2096 | 34 |
| ε3/ε4 | 178 | 460 | 89 | 509 | 3.20 [2.37; 4.33] | < 0.0001 | F | 0.5925 | 0 |
| ε4/ε4 | 37 | 460 | 8 | 509 | 5.21 [2.38; 11.37] | < 0.0001 | F | 0.5134 | 0 |
| **Ecuador** | **Numbers** | | | | **Association test** | |  | **Heterogeneity test** | |
| **Genotypes** | **Case** | | **Control** | | **OR[95% CI]** | **p** | **Model** | **p** | **I2 (%)** |
|  | **Events** | **Total** | **Events** | **Total** |  |  |  |  |  |
| ε2/ε2 | 0 | 95 | 0 | 97 | 5.42 [1.81; 16.24] | 0.0025 | F |  | NA |
| ε2/ε3 | 3 | 95 | 5 | 97 | 0.81 [0.19; 3.39] | 0.7739 | R | 0.1570 | 50 |
| ε2/ε4 | 2 | 95 | 1 | 97 | 1.04 [0.06; 16.98] | 0.9800 | F |  | NA |
| ε3/ε3 | 51 | 95 | 78 | 97 | 0.29 [0.20; 0.42] | < 0.0001 | F | 0.4723 | 0 |
| ε3/ε4 | 27 | 95 | 12 | 97 | 5.20 [2.27; 11.94] | < 0.0001 | F | 0.3815 | 0 |
| ε4/ε4 | 12 | 95 | 1 | 97 | 13.29 [3.62; 48.80] | < 0.0001 | F | 0.5671 | 0 |
| **Mexico** | **Numbers** | | | | **Association test** | |  | **Heterogeneity test** | |
| **Genotypes** | **Case** | | **Control** | | **OR[95% CI]** | **p** | **Model** | **p** | **I2 (%)** |
|  | **Events** | **Total** | **Events** | **Total** |  |  |  |  |  |
| ε2/ε2 | 19 | 418 | 6 | 497 | 0.28 [0.01; 5.88] | 0.4130 | F |  | NA |
| ε2/ε3 | 61 | 418 | 86 | 497 | 0.26 [0.14; 0.49] | < 0.0001 | F | 0.4293 | 0 |
| ε2/ε4 | 3 | 418 | 3 | 497 | 0.98 [0.23; 4.09] | 0.9762 | F | 0.3269 | 11 |
| ε3/ε3 | 221 | 418 | 358 | 497 | 0.55 [0.41; 0.73] | < 0.0001 | F | 0.1819 | 38 |
| ε3/ε4 | 84 | 418 | 40 | 497 | 2.60 [1.86; 3.63] | < 0.0001 | F | 0.2071 | 34 |
| ε4/ε4 | 30 | 418 | 4 | 497 | 5.57 [2.45; 12.69] | < 0.0001 | F | 0.9414 | 0 |
| **Peru** | **Numbers** | | | | **Association test** | |  | **Heterogeneity test** | |
| **Genotypes** | **Case** | | **Control** | | **OR[95% CI]** | **p** | **Model** | **p** | **I2 (%)** |
|  | **Events** | **Total** | **Events** | **Total** |  |  |  |  |  |
| ε2/ε2 | 0 | 79 | 0 | 128 | NA |  |  |  |  |
| ε2/ε3 | 3 | 79 | 8 | 128 | 1.89 [0.22; 16.29] | 0.5616 | * |  |  |
| ε2/ε4 | 0 | 79 | 0 | 128 | 0.08 [0.003; 2.13] | 0.1334 | * |  |  |
| ε3/ε3 | 43 | 79 | 102 | 128 | 0.23 [0.07; 0.74] | 0.0139 | * |  |  |
| ε3/ε4 | 28 | 79 | 17 | 128 | 5.02 [1.09; 23.12] | 0.0384 | * |  |  |
| ε4/ε4 | 5 | 79 | 1 | 128 | 3.70 [0.20; 68.37] | 0.3790 | * |  |  |
| **Venezuela** | **Numbers** | | | | **Association test** | |  | **Heterogeneity test** | |
| **Genotypes** | **Case** | | **Control** | | **OR[95% CI]** | **p** | **Model** | **p** | **I2 (%)** |
|  | **Events** | **Total** | **Events** | **Total** |  |  |  |  |  |
| ε2/ε2 | 0 | 309 | 5 | 1860 | 1.34 [0.14; 12.47] | 0.7946 | F | 0.6902 | 0 |
| ε2/ε3 | 14 | 309 | 153 | 1860 | 0.42 [0.12; 1.41] | 0.1603 | F | 0.9789 | 0 |
| ε2/ε4 | 9 | 309 | 27 | 1860 | 3.60 [0.25; 52.00] | 0.3477 | R | 0.1104 | 60 |
| ε3/ε3 | 159 | 309 | 1301 | 1860 | 0.37 [0.22; 0.62] | 0.0002 | F | 0.7388 | 0 |
| ε3/ε4 | 104 | 309 | 346 | 1860 | 2.13 [0.77; 5.91] | 0.1461 | R | 0.0924 | 58 |
| ε4/ε4 | 23 | 309 | 28 | 1860 | 7.76 [2.14; 28.09] | 0.0018 | F | 0.2985 | 17 |
| **Cuba + Jamaica** | **Numbers** | | | | **Association test** | |  | **Heterogeneity test** | |
| **Genotypes** | **Case** | | **Control** | | **OR[95% CI]** | **p** | **Model** | **p** | **I2 (%)** |
|  | **Events** | **Total** | **Events** | **Total** |  |  |  |  |  |
| ε2/ε2 | 0 | 95 | 0 | 80 | NA |  |  |  |  |
| ε2/ε3 | 8 | 95 | 9 | 80 | 1.32 [0.38; 4.58] | 0.6596 | F | 0.6752 | 0 |
| ε2/ε4 | 4 | 95 | 3 | 80 | 4.40 [0.48; 40.04] | 0.1889 | F | 0.7792 | 0 |
| ε3/ε3 | 44 | 95 | 45 | 80 | 0.27 [0.13; 0.56] | 0.0004 | F | 0.2597 | 21 |
| ε3/ε4 | 31 | 95 | 20 | 80 | 3.38 [0.98; 11.70] | 0.0542 | R | 0.1572 | 50 |
| ε4/ε4 | 8 | 95 | 3 | 80 | 2.49 [0.36; 17.31] | 0.3559 | F | 0.8073 | 0 |
